# Effect of *apolipoprotein E* ε4 and its modification by sociodemographic characteristics on cognitive measures in South Asians from LASI‐DAD

**DOI:** 10.1002/alz.14052

**Published:** 2024-06-18

**Authors:** Yi Zhe Wang, Wei Zhao, Priya Moorjani, Alden L. Gross, Xiang Zhou, Aparajit B. Dey, Jinkook Lee, Jennifer A. Smith, Sharon L. R. Kardia

**Affiliations:** ^1^ Department of Epidemiology School of Public Health University of Michigan Ann Arbor Michigan USA; ^2^ Survey Research Center Institute for Social Research University of Michigan Ann Arbor Michigan USA; ^3^ Department of Molecular and Cell Biology University of California Berkeley California USA; ^4^ Center for Computational Biology University of California Berkeley California USA; ^5^ Department of Epidemiology Johns Hopkins Bloomberg School of Public Health Baltimore Maryland USA; ^6^ Department of Biostatistics School of Public Health University of Michigan Ann Arbor Michigan USA; ^7^ Department of Geriatric Medicine All India Institute of Medical Sciences, Ansari Nagar New Delhi India; ^8^ Department of Economics and Center for Social Research University of Southern California Los Angeles California USA

**Keywords:** APOE, cognitive function, dementia, India, interaction, sociodemographic characteristics, South Asian

## Abstract

**BACKGROUND:**

We investigated the effects of *apolipoprotein E* (*APOE)* ε4 and its interactions with sociodemographic characteristics on cognitive measures in South Asians from the Diagnostic Assessment of Dementia for the Longitudinal Aging Study of India (LASI‐DAD).

**METHODS:**

Linear regression was used to assess the association between *APOE* ε4 and global‐ and domain‐specific cognitive function in 2563 participants (mean age 69.6 ± 7.3 years; 53% female). Effect modification by age, sex, and education were explored using interaction terms and subgroup analyses.

**RESULTS:**

*APOE* ε4 was inversely associated with most cognitive measures (*p* < 0.05). This association was stronger with advancing age for the Hindi Mental State Examination (HMSE) score (*β*_ε4×age _= −0.44, *p* = 0.03), orientation (*β*_ε4×age _= −0.07, *p* = 0.01), and language/fluency (*β*_ε4×age _= −0.07, *p* = 0.01), as well as in females for memory (*β*_ε4×male _= 0.17, *p* = 0.02) and language/fluency (*β*_ε4×male _= 0.12, *p* = 0.03).

**DISCUSSION:**

*APOE* ε4 is associated with lower cognitive function in South Asians from India, with a more pronounced impact observed in females and older individuals.

**Highlights:**

1. *APOE* ε4 carriers had lower global and domain‐specific cognitive performance.
2. Females and older individuals may be more susceptible to ε4 effects.
3. For most cognitive measures, there was no interaction between ε4 and education.

## 1 INTRODUCTION

Dementia, a group of neurological disorders characterized by cognitive impairment, is a leading cause of death, disability, and dependency among older adults worldwide.^1^, ^2^ Dementia and cognitive function have significant genetic components, with the ε4 allele of the *apolipoprotein E* (*APOE*) gene being the strongest genetic risk factor for late‐onset Alzheimer's disease (AD) in many populations.^3^, ^4^, ^5^ In European ancestry populations, having one ε4 allele is associated with an increase of 2‐3 times in the risk of AD compared to the most common and risk‐neutral ε3/ε3 genotype, while having two ε4 alleles is associated with an increase in risk of up to 15 times.^6^ Conversely, the ε2 allele is generally associated with reduced AD risk. These *APOE* alleles are also linked, to a lesser extent, with global cognition and specific cognitive domains including episodic memory, executive function, and verbal fluency in individuals without dementia.^7^

Sociodemographic factors such as age, sex, and education are also associated with cognitive function in older adults^8^ and contribute to the risk of dementia.^9^, ^10^ There is increasing evidence that these factors interact with *APOE* ε4 on cognitive function and dementia. Some studies suggest that the ε4 effect on AD may be more pronounced in young‐old adults (e.g., 60‐70 years) compared to older individuals (> 70 years),^6^, ^11^, ^12^ in females compared to males,^6^, ^13^, ^14^, ^15^ and in those with lower education.^16^, ^17^ Similar interactions of ε4 with sex and education are observed with cognitive function as the outcome.^18^, ^19^, ^20^, ^21^ In contrast to AD, the ε4 effect on cognitive function appears to increase progressively with age.^22^, ^23^ However, a few studies have not found significant interactions between *APOE* ε4 and age, sex, or education in relation to cognitive function and dementia.^24^, ^25^, ^26^, ^27^

To date, genomic studies on cognitive function and dementia have predominantly focused on European ancestry populations.^28^, ^29^ India is the most populous country in the world with over 4.1 million people estimated to have dementia,^2^ yet Indian and South Asian populations in general are rarely represented in genomic studies. This underrepresentation is concerning as genetic effects and disease associations could vary across ancestry groups due to differences in allele frequencies and linkage disequilibrium patterns.^30^ Indeed, some evidence suggests that the effect of *APOE* ε4 on cognitive function and dementia differs across ancestries and race/ethnicities.^31^ Previous studies showed that the association between ε4 and AD was weaker among African Americans and Hispanics compared to Caucasians, but stronger in Japanese.^6^ Other studies investigating the association of ε4 with cognitive function and decline also reported a weaker effect among African Americans and Hispanics.^32^, ^33^ In India, the few studies that have investigated the relationship between *APOE* ε4 and AD suggest that its effect size is similar to that observed in populations of European ancestry.^34^, ^35^, ^36^, ^37^ However, these studies have primarily relied on geographically restricted samples within India. Furthermore, unique cultural, social, and environmental characteristics of India, such as dietary practices and pathogen or toxicant exposures, may be associated with cognitive function and dementia and/or modulate the genetic effect of *APOE* ε4 in various ways. Therefore, large‐scale epidemiological studies employing nationally representative data are needed to comprehensively explore the effects of *APOE* ε4 on cognition and dementia and identify potential effect modifiers in Indian/South Asian populations.

The Diagnostic Assessment of Dementia for Longitudinal Aging Study in India (LASI‐DAD) is a population‐based prospective cohort study that has collected nationally representative data on Indians aged 60 and older to better understand the determinants of late‐life cognition, cognitive aging, and dementia. In this study, we examined the effect of the *APOE* ε4 allele and its potential modification by sociodemographic factors including age, sex, and education on cognitive measures in LASI‐DAD. Based on prior literature, we hypothesize that the association between *APOE* ε4 and cognitive function would be more pronounced in older individuals, females, and those with a lower education level.

**RESEARCH IN CONTEXT**

1. **Systematic review**: We reviewed the literature using traditional sources (e.g., PubMed). There were several studies examining age, sex, and education as potential effect modifiers of the association between *apolipoprotein E* (*APOE)* ε4 and cognitive function in populations of European ancestry, which are properly cited. However, it is unclear whether ε4 has similar effects in Indian/South Asian populations. Existing studies in this population focused on Alzheimer's disease (AD) and primarily relied on geographically restricted, non‐representative samples of India.
2. **Interpretation**: In a nationally representative sample of older Indians, *APOE* ε4 is adversely associated with cognitive function, especially among women and older individuals.
3. **Future directions**: Future large‐scale longitudinal research in the Indian/South Asian population is warranted to replicate our findings and further evaluate the age‐, sex‐, and education‐related changes in the effects of *APOE* ε4 on cognitive aging.

## 2 METHODS

### 2.1 Study population

The Longitudinal Aging Study of India (LASI) is a nationally representative sample of more than 73,000 adults from India aged 45 years or older.^38^ LASI‐DAD is an add‐on study of late‐life cognition and dementia, in which a subsample of approximately 4000 community‐residing LASI respondents aged 60 or older were administered in‐depth cognitive tests and informant interviews. A two‐stage stratified random sampling method was used in LASI‐DAD to ensure a sample with a broad distribution of cognitive ability including respondents with dementia and mild cognitive impairment (MCI). The respondents were stratified according to their state of residence and their risk of cognitive impairment based on their performance on the cognitive tests and proxy interview (i.e., interview with a family member when the respondent is incapable of participating in the interview) in the main LASI study. Next, within each state, a predetermined sample size proportional to the state's population size was used to randomly select an equal number of respondents at high and low risk of cognitive impairment.^39^ The baseline (Wave 1) LASI‐DAD sample consists of 4096 older adults aged 60 or older, representing > 600 ethnically and geographically diverse areas within 18 states and union territories.

### 2.2 Measures

#### 2.2.1 Whole genome sequencing (WGS)

WGS was performed on a total of 2762 individuals at MedGenome, Inc (Bangalore, India) at an average read depth of 30. After removing the control samples, a total of 2736 LASI‐DAD participants who consented to blood sample collection had WGS data available for analysis. There is minimal evidence of significant differences in cognitive function between participants who underwent WGS and those who did not (Table S1). Genotype calling and quality control (QC) on the raw LASI‐DAD WGS data were performed at the Genome Center for Alzheimer's Disease (GCAD) at the University of Pennsylvania.^40^, ^41^ Briefly, sample‐level QC included checks for low coverage, sample contamination, sex discrepancies, concordance with previous genotype data, and duplicates.^40^ After further excluding samples with low quality and/or unresolved identity, a total of 2680 samples were retained in the analysis. At the genotype level, each genotype was evaluated and set to missing if read depth was less than 10 or genotype quality score was less than 20. At the variant level, a variant was excluded if it was monomorphic, was above the 99.8% Variant Quality Score Recalibration (VQSR) Tranche (the quality score was beyond the range that contains 99.8% of true variants), had a call rate ≤ 80%, or had an average mean depth > 500 reads. We further removed variants that were in low complexity regions identified with mdust.^42^ After QC and filtering, we retained a total of 71,109,961 autosomal bi‐allelic variants that include 66,204,161 single nucleotide polymorphisms (SNPs) and 4,905,800 indels. Genetic principal components (PCs) were calculated using PCAir^43^ to take into account the relatedness in the samples. Specifically, the PCs were first calculated in a set of unrelated individuals (kinship cutoff = 0.044) and then projected to all other individuals. For this analysis, we included 2607 individuals that are not related at 3rd degree or above. The top 10 PCs were included in all analyses to adjust for population stratification.

#### 2.2.2 *APOE* genotyping

*APOE* ε2, ε3, and ε4 alleles are defined by two SNPs (rs429358 and rs7412) that lead to changes in the amino acid sequence at positions 112 and 158. Direct genotyping for these *APOE* SNPs was performed on 2754 LASI‐DAD participants using TaqMan assays (Applied Biosystems, Foster City, CA, USA). The resulting genotypes of these two SNPs were then combined to form six possible *APOE* genotypes: ε2/ε2, ε2/ε3, ε2/ε4, ε3/ε3, ε3/4, and ε4/ε4. In our primary analyses, we dichotomized *APOE* genotypes based on the presence or absence of the ε4 alleles and classified participants as *APOE* ε4 carriers (i.e., ε3/4, and ε4/ε4) or noncarriers (i.e., ε2/ε2, ε2/ε3, and ε3/ε3). We focused on the 2590 unrelated participants who had both WGS and *APOE* genotyping data. Due to the potential opposing effects of the ε4 and ε2 alleles on cognitive measures, individuals with the *APOE* ε2/ε4 genotype were further excluded from the analyses (*N* = 27, 1.0%). This left 2563 participants for inclusion in the primary analyses. Of these, 1828 (71.3%) participants were considered cognitively intact, while 550 (21.5%) were classified with mild neurocognitive disorder, and 185 (7.2%) with major neurocognitive disorder, based on a diagnostic algorithm using the Diagnostic and Statistical Manual of Mental Disorders, Fifth Edition (DSM‐5) criteria.^44^ A study flow chart is presented in Figure S1.

#### 2.2.3 Cognitive measures

LASI‐DAD administered a detailed battery of cognitive tests to measure a range of key cognitive domains affected by cognitive aging including memory, processing speed, language, executive function, and visuospatial skills, closely following the Harmonized Cognitive Assessment Protocol (HCAP). The HCAP was developed for the assessment of dementia and MCI in the US Health and Retirement Study and its sister studies around the world to enable international comparisons. In LASI‐DAD, appropriate modifications were made to the HCAP to reflect India's unique cultural and contextual constraints. Notably, to accommodate the considerable proportion of older Indian adults who are illiterate and/or innumerate, the selection and administration of specific cognitive tests were adapted to reduce the dependency on literacy and numeracy skills.^39^, ^45^ Cognitive tests were evaluated through two pretests conducted prior to the first wave of data collection. Further adjustments were made to the protocol based on the observations and feedback received from these two pretests.^39^, ^45^ All cognitive tests were validated in India.

Cognitive testing was carried out in the mother tongue of LASI‐DAD participants. The cognitive tests were translated from English into 12 local languages, including Hindi, Kannada, Malayalam, Gujarati, Tamil, Punjabi, Urdu, Bengali, Assamese, Odiya, Marathi, and Telugu, employing a rigorous forward and backward translation approach. Gross et al. evaluated the measurement invariance of the LASI‐DAD cognitive test battery across languages of administration and found no statistically significant evidence of measurement differences associated with administration language.^46^

We examined a total of seven cognitive measures, including the Hindi Mental State Examination (HMSE) score, five broad cognitive domain scores (orientation, memory, executive functioning, language/fluency, and visuospatial), and a general cognitive function summary score constructed from the five domains. The HMSE score is the Hindi version of the Mini‐Mental State Examination (MMSE), a widely used cognitive screening instrument.^47^ The HMSE score ranges from 0 to 30, with higher scores indicating better cognitive functioning. General cognitive function and domain‐specific cognitive performance were derived using factor analysis of the LASI‐DAD cognitive battery, as described by Gross et al.^46^ Briefly, cognitive tests were classified into broad domains and further divided into narrow subdomains based on a priori knowledge and theory, following the structure of the Cattell‐Horn‐Carroll (CHC) theory of human cognitive abilities. The specific cognitive tests included in each domain are listed in Table S2. Unidimensional factor analyses were conducted separately for each narrow and broad cognitive domain. Once an adequate fit was achieved for each model, all the domains were combined into a hierarchical multiple domain factor analysis that included a general factor. The resulting factor scores were internally standardized to a *N*(0,1) distribution in the entire sample of 4096 LASI‐DAD participants.

#### 2.2.4 Covariates and effect modifiers

We examined several sociodemographic and socioeconomic characteristics that were self‐reported by the participants at baseline as covariates. Sociodemographic factors included age, sex, educational level according to a three‐tier simplified version of the 1997 International Standard Classification of Education (ISCED‐97) codes (less than secondary education, upper secondary education and vocational training, or tertiary education), urban/rural residence, literacy (can read or write), and state of residence. We also included caste and per capita household consumption (in rupees) as measures of socioeconomic status. Respondents self‐reported their caste and were classified into four official categories recognized by the Indian government: scheduled caste, scheduled tribe, other backward class (OBC), or no caste/other caste. Per capita household consumption was used to capture the economic status of the respondents and was calculated by taking total household consumption over the previous year for food, household utilities, fees, durable goods, education, healthcare, discretionary spending, transit, and remittances divided by the number of people in the household. In cases of missing data, which were limited to two covariates (caste and per capita household consumption) in our main analyses and affected fewer than 0.6% of the study sample, we conducted a complete case analysis by excluding those individuals from the model.

Age, sex, and education were also assessed as potential effect modifiers of the association between *APOE* ε4 and cognitive function. Effect modification by age was tested using interaction terms between ε4 and continuous age. We also examined ε4 effects in stratified analysis by 5‐year age group, with those aged 85 and older combined into a single group. We examined the interaction between ε4 and education using the three‐level categorical education variable described above. Nearly half of our sample (∼48.7%) had never attended school, which aligns with the 56.5% illiteracy rate among older adults in India.^48^ Thus, we further investigated the interaction between *APOE* ε4 and a dichotomous education variable, distinguishing between individuals with no formal education and those with any formal education (0 years of education vs. > 0 years of education groups).

### 2.3 Statistical analysis

The distribution of *APOE* genotypes and allele frequencies were calculated for the full study sample, and Hardy‐Weinberg equilibrium was assessed. *APOE* genotype and allele frequencies were additionally estimated by 5‐year age group ranging from 60 years (smallest age of the sample) to ≥ 85 years. We compared and tested whether the *APOE* ε4 allele frequency differed by age to assess a potential selective survival bias. Descriptive statistics of the analytic sample were compared by *APOE* ε4 carrier status using *t*‐test, Wilcoxon rank‐sum test, and Pearson chi‐squared test as appropriate.

We first examined the association between *APOE* ε4 (carrier vs. non‐carrier) and cognitive measures in the full analytic sample using linear regression models. We then tested the interaction between *APOE* ε4 and age, sex, or education by including the two independent variables and their cross‐product term into the same model. We further conducted age‐, sex‐, and education‐stratified analyses to examine the ε4 effects separately within each subgroup, regardless of whether an interaction was detected. We employed two sets of models for all analyses. Model 1 adjusted for age (if applicable), sex (if applicable), state of residence, and the top 10 genetic PCs. Model 2 additionally adjusted for social‐ and economic‐related variables including educational level (if applicable), literacy, urban/rural residence, caste, and per capita household consumption.

We performed two sensitivity analyses. First, we repeated our analyses to examine the effects of specific *APOE* genotypes and their modification by age, sex, and education on cognitive measures. In this analysis, we excluded individuals with the ε2/ε4 (protective/risk allele) and ε2/ε2 (low frequency; *N* = 5) genotypes and focused on the remaining four possible *APOE* genotypes: ε3/ε3 (reference group), ε2/ε3, ε3/ε4, and ε4/ε4. However, due to small subgroup sample sizes, we did not estimate the effect of *APOE* genotype within 5‐year age groups or assess the *APOE* genotype interaction with the three‐tier education variable and its subgroup effects. Second, there is growing evidence that vascular risk factors, including hypertension, diabetes, hypercholesterolemia, obesity, and smoking, increase the risks of cognitive decline and dementia.^49^ In India, despite the high prevalence of these risk factors, their impact on cognitive function remains unclear.^50^ Therefore, for any identified ε4 effects and interactions, we additionally controlled for self‐reported physician diagnosis of high blood pressure, high cholesterol, diabetes, and smoking (ever smoked), as well as measured body mass index (categorized as underweight, normal weight, overweight/obese) in a subset of individuals who had complete data for these variables (*N* = 2351) to address potential confounding from cardiovascular risk factors. For all analyses, statistical significance was established at *p*‐value < 0.05. Given that all our hypotheses were pre‐defined and grounded in priori evidence, and considering the inherently correlated nature of cognitive measures, we did not correct for multiple testing in this study.[Table alz14052-tbl-0001]

## 3 RESULTS

### 3.1 Sample characteristics

Table S3 provides the distribution of *APOE* genotypes and alleles in the full study population, as well as by 5‐year age intervals ranging from 60 years to ≥ 85 years. The allele frequencies of *APOE* ε2, ε3, and ε4 were 0.048, 0.844, and 0.108, respectively. There was no significant difference in the frequency of the ε4 allele by age when compared across 5‐year age groups (*p* = 0.813) (Table S3 and Figure S2). About 19% (*N* = 497) of the participants were classified as *APOE* ε4 carriers (18% ε3/ε4 heterozygotes and 1% ε4/ε4 homozygotes). Genotype frequencies observed in the full study sample were in Hardy‐Weinberg equilibrium (*p* = 0.667).

Descriptive statistics for sociodemographic characteristics and cognitive measures by *APOE* ε4 carrier status are shown in Table 1. The mean age of the participants was 69.56 years (SD 7.29) and approximately 53% (*N* = 1358) were female. Seventy‐five percent (*N* = 1926) had less than lower secondary education, with 48.7% (*N* = 1249) participants having no formal education. Twenty‐one percent (*N* = 542) had upper secondary or vocational training, and 4% (*N* = 95) had tertiary education. *APOE* ε4 carriers and noncarriers did not differ with respect to age, sex, and state of residence. However, compared to *APOE* ε4 noncarriers, ε4 carriers were more likely to have less than upper secondary education, reside in rural areas, and score lower on all cognitive measures except visuospatial functioning. Significant differences in caste and per capita household consumption were also found between *APOE* ε4 carriers and noncarriers. Table S4 provides additional descriptive statistics for vascular risk factors. Compared to noncarriers, ε4 carriers were more likely to be underweight. Distribution of other vascular risk factors did not significantly differ between the two groups.

**TABLE 1 alz14052-tbl-0001:** Descriptive statistics by *APOE* ε4 carrier status among 2563 participants in LASI‐DAD.

	Total (*N* = 2563)	*APOE* ε4 noncarrier (*N* = 2066)	*APOE* ε4 carrier (*N* = 497)	*p* value
Age, mean (SD), years	69.56 (7.29)	69.55 (7.27)	69.59 (7.39)	0.996^a^
Female sex	1358 (53.0%)	1098 (53.1%)	260 (52.3%)	0.777^b^
State of residence				0.084^b^
Assam	68 (2.7%)	47 (2.3%)	21 (4.2%)	
Bihar	129 (5.0%)	101 (4.9%)	28 (5.6%)	
Delhi	119 (4.6%)	94 (4.5%)	25 (5.0%)	
Gujarat	220 (8.6%)	174 (8.4%)	46 (9.3%)	
Haryana	165 (6.4%)	137 (6.6%)	28 (5.6%)	
Jammu and Kashmir	85 (3.3%)	73 (3.5%)	12 (2.4%)	
Karnataka	115 (4.5%)	98 (4.7%)	17 (3.4%)	
Kerala	250 (9.8%)	208 (10.1%)	42 (8.5%)	
Madhya Pradesh	73 (2.8%)	59 (2.9%)	14 (2.8%)	
Maharashtra	173 (6.7%)	131 (6.3%)	42 (8.5%)	
Orissa	206 (8.0%)	156 (7.6%)	50 (10.1%)	
Punjab	105 (4.1%)	83 (4.0%)	22 (4.4%)	
Rajasthan	144 (5.6%)	124 (6.0%)	20 (4.0%)	
Tamil Nadu	155 (6.0%)	125 (6.1%)	30 (6.0%)	
Telangana	164 (6.4%)	142 (6.9%)	22 (4.4%)	
Uttar Pradesh	148 (5.8%)	114 (5.5%)	34 (6.8%)	
Uttaranchal	63 (2.5%)	51 (2.5%)	12 (2.4%)	
West Bengal	181 (7.1%)	149 (7.2%)	32 (6.4%)	
Education				0.024^b^
Less than lower secondary	1926 (75.1%)	1529 (74.0%)	397 (79.9%)	
Upper secondary or vocational training	542 (21.1%)	458 (22.2%)	84 (16.9%)	
Tertiary	95 (3.7%)	79 (3.8%)	16 (3.2%)	
Education (dichotomous)				0.067^b^
No formal education (0 years of education)	1249 (48.7%)	988 (47.8%)	261 (52.5%)	
Some level of education (> 0 years of education)	1314 (51.3%)	1078 (52.2%)	236 (47.5%)	
Rural residence	1625 (63.4%)	1283 (62.1%)	342 (68.8%)	0.006^b^
Illiteracy (cannot read or write)	1455 (56.8%)	1157 (56.0%)	298 (60.0%)	0.121^b^
Caste (*N* = 2550)				0.001^b^
Scheduled caste	467 (18.2%)	376 (18.2%)	91 (18.3%)	
Scheduled tribe	99 (3.9%)	66 (3.2%)	33 (6.6%)	
Other backward class	1120 (43.7%)	896 (43.4%)	224 (45.1%)	
No caste or other caste	864 (33.7%)	717 (34.7%)	147 (29.6%)	
Per capita household consumption in quintiles, rupees (*N* = 2561)				0.013^b^
First (≤ 21,973)	512 (20.0%)	388 (18.8%)	124 (24.9%)	
Second (21,973‐30,820)	514 (20.1%)	416 (20.1%)	98 (19.7%)	
Third (30,820‐42,489)	509 (19.9%)	406 (19.7%)	103 (20.7%)	
Fourth (42,489‐62,668)	514 (20.1%)	431 (20.9%)	83 (16.7%)	
Fifth (≥ 62,668)	512 (20.0%)	423 (20.5%)	89 (17.9%)	
HMSE score, mean (SD)	22.69 (5.38)	22.90 (5.17)	21.79 (6.08)	0.002^a^
General cognitive function, mean (SD)	0.005 (0.91)	0.036 (0.90)	−0.124 (0.98)	0.001^c^
Cognitive domain scores, mean (SD)				
Orientation	−0.025 (0.79)	0.003 (0.78)	−0.143 (0.85)	4.84E‐04^c^
Executive function	−0.006 (0.9)	0.019 (0.89)	−0.112 (0.92)	0.004^c^
Language/fluency	−0.032 (0.8)	−0.006 (0.78)	−0.138 (0.88)	0.002^c^
Memory	0.021 (0.93)	0.053 (0.93)	−0.112 (0.96)	0.001^c^
Visuospatial function	0.029 (0.83)	0.039 (0.82)	−0.008 (0.84)	0.261^c^

*Note*: Individuals with the *APOE* ε2/ε4 genotype were excluded from the sample. *N* (%) are reported unless otherwise specified.

Abbreviations: *APOE*, apolipoprotein E; HMSE, Hindi mental state examination; LASI‐DAD, diagnostic assessment of dementia for the longitudinal aging study of India; SD, standard deviation.

^a^ *p*‐value calculated from Wilcoxon rank sum test.

^b^ *p*‐value calculated from Chi‐square test.

^c^ *p*‐value calculated from *t*‐test.

Table S5 provides the correlation among the seven cognitive measures examined in this study. The correlation among the five cognitive domain scores ranged from 0.491 to 0.733. The correlations between the five domain scores and general cognitive function and HMSE ranged from 0.733 to 0.948 and from 0.560 to 0.856, respectively.

### 3.2 *APOE* ε4 and sociodemographic characteristic associations with cognitive measures

Table 2 presents the associations between *APOE* ε4 and cognitive measures in the full sample. All effect estimations were in the expected direction, with ε4 carriers having lower cognitive function. In models adjusting for age, sex, state of residence, and the top 10 genetic PCs (Model 1), ε4 carrier status was associated with all cognitive measures except visuospatial functioning (*p* < 0.05). Specifically, being an ε4 carrier was associated with a 0.095‐0.142 SD decrease in cognitive scores and a 0.978 points reduction in HMSE score. The associations were slightly attenuated after further controlling for education, literacy, urban/rural residence, caste, and per capita household consumption in Model 2. Nevertheless, the association between *APOE* ε4 and HMSE score, general cognitive function, orientation, and memory remained significant (*p* < 0.05).

**TABLE 2:** Effects of *APOE* ε4 carrier status on cognitive measures in LASI‐DAD.

	Model 1 (*N* = 2563)			Model 2 (*N* = 2548)		
Cognitive measure	Beta	*p* value	ΔR^2^	Beta	*p* value	ΔR^2^
**HMSE score**	**−0.978**	**2.44E‐05**	0.51%	**−0.710**	**0.001**	0.27%
**General cognitive function**	**−0.142**	**1.69E‐04**	0.37%	**−0.079**	**0.006**	0.11%
**Executive function**	**−0.117**	**0.002**	0.26%	−0.057	0.054	0.06%
**Orientation**	**−0.141**	**2.86E‐05**	0.49%	**−0.096**	**0.001**	0.22%
**Language/fluency**	**−0.095**	**0.005**	0.21%	−0.052	0.064	0.07%
**Memory**	**−0.134**	**0.001**	0.32%	**−0.087**	**0.019**	0.13%
**Visuospatial function**	−0.063	0.097	0.09%	−0.019	0.576	0.01%

*Note*: Model 1 adjusted for age, sex, state of residence, and the top 10 genetic PCs.

Model 2 adjusted for age, sex, state of residence, top 10 genetic PCs, education (less than lower secondary education, upper secondary or vocational training and tertiary education), literacy, urban/rural residence, caste, and quintiles of per capita household consumption.

Δ*R* ^2^ represents the change in *R* ^2^ when *APOE* ε4 carrier status was added to the corresponding regression models.

Beta coefficient and *p*‐value in bold indicates statistically significant association at *p* < 0.05.

Abbreviations: APOE, apolipoprotein E; HMSE, Hindi mental state examination; LASI‐DAD, diagnostic assessment of dementia for the longitudinal aging study of India; PCs, principal components.

Effect estimates and *p*‐values for all other covariates in Models 1 and 2 can be found in Tables S6 and S7, respectively. In Model 1, older age was inversely associated with all cognitive measures, while male sex was positively associated with all cognitive measures. These associations stayed largely consistent in Model 2. Education was also independently associated with all cognitive measures. Compared to individuals with less than lower secondary education, those with upper secondary or vocational training education showed a 0.179‐0.458 SD increase in cognitive scores and a 1.480 point increase in HMSE score, while those with tertiary education showed a 0.327‐0.893 SD increase in cognitive scores and a 2.392 points increase in HMSE score.

Collectively, *APOE* ε4 carrier status and other covariates in Model 1 explained approximately 18.6% (visuospatial function) to 33.9% (general cognitive function) of the total variance in cognitive measures. Specifically, ε4 carrier status alone contributed between 0.09% (visuospatial function) and 0.51% (HMSE score) of the explained variance. The total variance explained increased to 37.9%‐62.4% when education, literacy, urban/rural residence, caste, and per capita household consumption were added to the model (Model 2). In this further adjusted model, the individual contribution of ε4 carrier status to the total variance explained ranged from 0.01% to 0.27%.

### 3.3 Effect modification of age, sex, and education on the associations between *APOE* ε4 and cognitive measures

**FIGURE 1:**
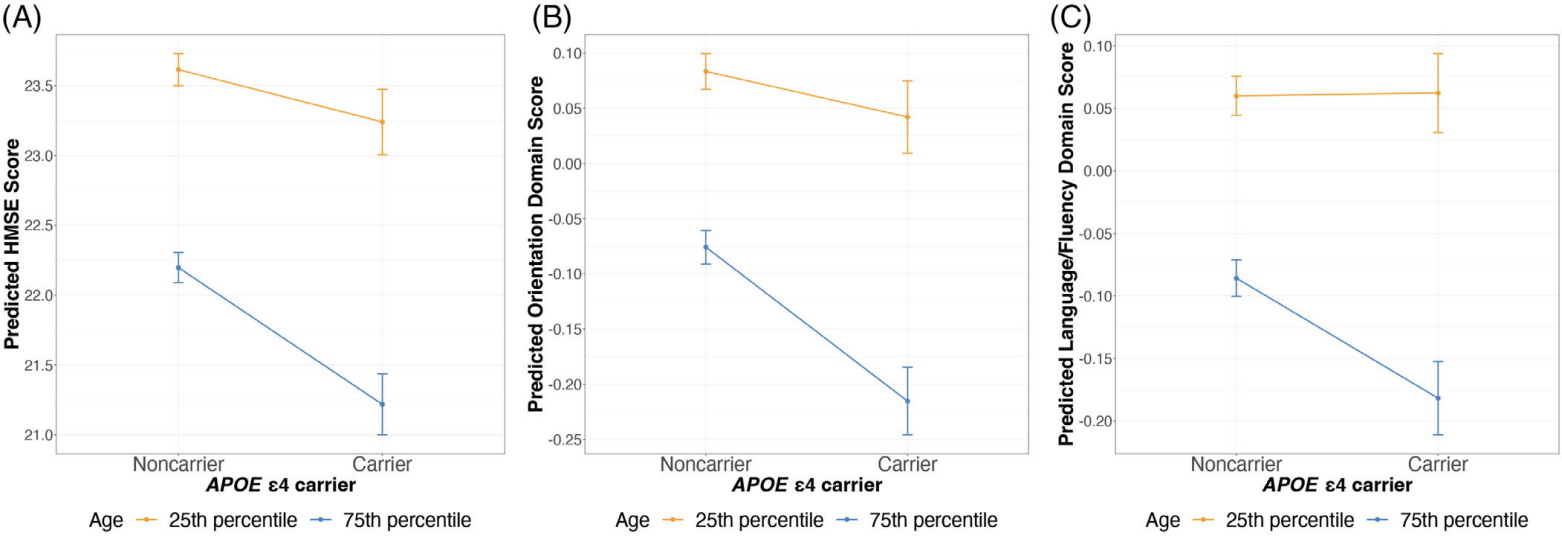
Plots of significant two‐way interactions between apolipoprotein E (*APOE)* ε4 and age on cognitive measures. Predicted HMSE score (A), orientation score (B), and language/fluency score (C) of *APOE* ε4 carriers and noncarriers at the 25th (64 years) and 75th percentile (74 years) of age are shown with standard error bars by age group. Cognitive measures are predicted using models adjusted for age, sex (male), state of residence, top 10 genetic PCs, education (less than lower secondary, upper secondary or vocational training and tertiary education), urban/rural residence, literacy, caste, quintiles of per capita household consumption, and *APOE* ε4 × age (Model 2). Color coding is according to age. *APOE*, apolipoprotein E; HMSE, Hindi‐mini‐mental state examination; PCs, principal components.

We next examined whether the associations of *APOE* ε4 with cognitive measures were modified by age, sex, and education. Table 3 provides the results from testing the *APOE* ε4 × age interaction term. We found a significant *APOE* ε4 by age interaction on HMSE score, orientation, and language/fluency in Model 2, with the associations being more pronounced with advancing age. To illustrate the identified age interactions, Figure 1 shows the predicted cognitive scores for ε4 carriers and noncarriers at the 25th (64 years) and 75th percentile (74 years) of age. Examining the ε4 effect across 5‐year age groups demonstrates a similar trend, where there is an overall increase in the magnitude of the ε4 effect as age increases, particularly from the 70‐75 age group onward, culminating in the strongest association identified in the ≥ 85 age group. This trend was consistent across most cognitive measures, except for visuospatial function, where the ε4 effect was most pronounced in the 60‐65 age group (Figure S3). However, due to the small sample sizes in each 5‐year age group, we note that while the trend appears to show increased effects at older ages, most effect estimates are not significantly different from zero.

**TABLE 3:** Two‐way interaction between *APOE* ε4 carrier status and age on cognitive measures.

	Model 1 (*N* = 2563)		Model 2 (*N* = 2548)	
	Beta	*p* value	Beta	*p* value
**HMSE score** *				
*APOE* ε4	**−0.977**	**2.46E‐05**	**−0.710**	**0.001**
Age	**−0.175**	**2.11E‐34**	**−0.142**	**1.61E‐27**
*APOE* ε4 × Age	−0.348	0.127	**−0.441**	**0.032**
**General cognitive function**				
*APOE* ε4	**−0.142**	**1.70E‐04**	**−0.079**	**0.006**
Age	**−0.033**	**4.10E‐46**	**−0.025**	**1.42E‐42**
*APOE* ε4 × Age	−0.024	0.527	−0.051	0.071
**Executive function**				
*APOE* ε4	**−0.117**	**0.002**	−0.057	0.055
Age	**−0.030**	**3.35E‐38**	**−0.021**	**1.90E‐30**
*APOE* ε4 × Age	−0.010	0.780	−0.041	0.165
**Orientation** *				
*APOE* ε4	**−0.141**	**2.88E‐05**	**−0.096**	**0.001**
Age	**−0.021**	**1.12E‐24**	**−0.016**	**2.08E‐18**
*APOE* ε4 × Age	−0.058	0.080	**−0.072**	**0.013**
**Language/fluency** *				
*APOE* ε4	**−0.094**	**0.005**	−0.052	0.064
Age	**−0.020**	**1.01E‐22**	**−0.015**	**6.79E‐17**
*APOE* ε4 × Age	−0.059	0.075	**−0.072**	**0.010**
**Memory**				
*APOE* ε4	**−0.134**	**0.001**	**−0.087**	**0.019**
Age	**−0.037**	**1.11E‐46**	**−0.030**	**1.16E‐38**
*APOE* ε4 × Age	0.006	0.886	−0.016	0.660
**Visuospatial function**				
*APOE* ε4	−0.063	0.097	−0.019	0.576
Age	**−0.023**	**1.85E‐23**	**−0.016**	**4.28E‐15**
*APOE* ε4 × Age	0.016	0.670	−0.007	0.831

*Note*: Model 1 adjusted for age (continuous and centered at the sample mean), sex (male), state of residence, top 10 genetic PCs, and *APOE* ε4 × Age.

Model 2 adjusted for age (continuous and centered at the sample mean), sex (male), state of residence, top 10 genetic PCs, education (less than lower secondary education, upper secondary or vocational training and tertiary education), literacy, urban/rural residence, caste, quintiles of per capita household consumption, and *APOE* ε4 × Age.

Beta coefficient and *p*‐value in bold indicates statistically significant association at *p* < 0.05.

Asterisk (*) denotes cognitive measures for which a statistically significant interaction term was observed.

Abbreviations: APOE, apolipoprotein E; HMSE, Hindi mental state examination; LASI‐DAD, diagnostic assessment of dementia for the longitudinal aging study of India; PCs, principal components.

**FIGURE 2:**
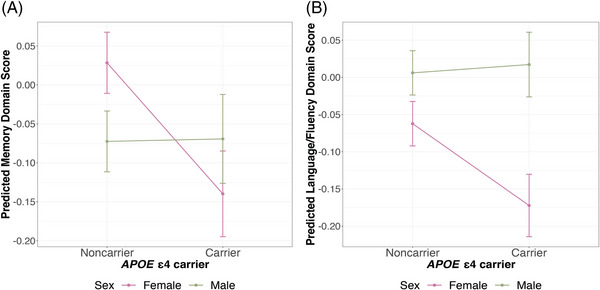
Plots of significant two‐way interactions between *APOE* ε4 and sex on cognitive measures. Predicted memory score (A) and language/fluency score (B) of *APOE* ε4 carriers and noncarriers are shown with standard error bars by sex. Cognitive measures are predicted using models adjusted for age, sex (male), state of residence, top 10 genetic PCs, education (less than lower secondary, upper secondary or vocational training, and tertiary education), urban/rural residence, literacy, caste, quintiles of per capita household consumption, and *APOE* ε4 × male (Model 2). Color coding is according to sex. *APOE*, apolipoprotein E; PCs, principal components.

Table 4 presents the results from testing the *APOE* ε4 by sex interaction term. In Model 1, an *APOE* ε4 × male interaction was observed for language/fluency and memory, with the effect of ε4 stronger in females. While the interaction terms were not statistically significant for the other cognitive measures, sex‐stratified analysis showed that *APOE* ε4 was associated with all cognitive measures among females except visuospatial functioning, whereas there was no association between ε4 and any cognitive measures among males (Table 5). Sex differences in the ε4 effect attenuated in Model 2, but the *APOE* ε4 × male sex interaction term remained statistically significant for both language/fluency and memory (Figure 2, Tables 4 and 5).

**TABLE 4:** Two‐way interaction between *APOE* ε4 carrier status and sex on cognitive measures.

	Model 1 (*N* = 2563)		Model 2 (*N* = 2548)	
	Beta	*p* value	Beta	*p* value
**HMSE score**				
*APOE* ε4	**−1.334**	**3.00E‐05**	**−0.997**	**0.001**
Male	**3.196**	**9.87E‐53**	**1.696**	**1.94E‐17**
*APOE* ε4 × Male	0.748	0.105	0.603	0.149
**General cognitive function**				
*APOE* ε4	**−0.199**	**1.34E‐04**	**−0.121**	**0.002**
Male	**0.611**	**1.53E‐70**	**0.232**	**2.20E‐17**
*APOE* ε4 × Male	0.120	0.112	0.089	0.121
**Executive function**				
*APOE* ε4	**−0.128**	**0.014**	−0.056	0.177
Male	**0.637**	**6.23E‐77**	**0.287**	**7.69E‐24**
*APOE* ε4 × Male	0.023	0.762	−0.004	0.951
**Orientation**				
*APOE* ε4	**−0.203**	**1.26E‐05**	**−0.145**	**3.50E‐04**
Male	**0.628**	**2.72E‐91**	**0.378**	**6.79E‐41**
*APOE* ε4 × Male	0.130	0.053	0.103	0.079
**Language/fluency** *				
*APOE* ε4	**−0.166**	**3.88E‐04**	**−0.110**	**0.005**
Male	**0.355**	**8.35E‐32**	**0.068**	**0.010**
*APOE* ε4 × Male	**0.149**	**0.027**	**0.121**	**0.031**
**Memory** *				
*APOE* ε4	**−0.223**	**8.44E‐05**	**−0.168**	**0.001**
Male	**0.172**	**2.09E‐06**	**−0.101**	**0.004**
*APOE* ε4 × Male	**0.185**	**0.024**	**0.171**	**0.020**
**Visuospatial function**				
*APOE* ε4	−0.088	0.091	−0.033	0.471
Male	**0.441**	**1.92E‐38**	**0.148**	**2.66E‐06**
*APOE* ε4 × Male	0.054	0.478	0.030	0.647

*Note*: Model 1 adjusted for age, sex (male), state of residence, top 10 genetic PCs, and *APOE* ε4 × male.

Model 2 adjusted for age, sex (male), state of residence, top 10 genetic PCs, education (less than lower secondary education, upper secondary or vocational training and tertiary education), literacy, urban/rural residence, caste, quintiles of per capita household consumption, and *APOE* ε4 × male.

Beta coefficient and *p*‐value in bold indicates statistically significant association at *p* < 0.05.

Asterisk (*) denotes cognitive measures for which a statistically significant interaction term was observed.

Abbreviations: APOE, apolipoprotein E; HMSE, Hindi mental state examination; LASI‐DAD, diagnostic assessment of dementia for the longitudinal aging study of India; PCs, principal components.

**TABLE 5:** Associations between *APOE* ε4 carrier status and cognitive measures stratified by sex.

	Model 1				Model 2			
	Male (*N* = 1205)		Female (*N* = 1358)		Male (*N* = 1200)		Female (*N* = 1348)	
**Cognitive measure**	**Beta**	***p* value**	**Beta**	***p* value**	**Beta**	***p* value**	**Beta**	***p* value**
**HMSE score**	−0.522	0.105	**−1.249**	**1.72E‐04**	−0.395	0.171	**−0.904**	**0.003**
**General cognitive function**	−0.072	0.202	**−0.187**	**2.09E‐04**	−0.037	0.386	**−0.113**	**0.004**
**Executive function**	−0.098	0.091	**−0.118**	**0.016**	−0.060	0.186	−0.052	0.191
**Orientation**	−0.056	0.235	**−0.203**	**2.16E‐05**	−0.039	0.340	**−0.144**	**0.001**
**Language/fluency** *	−0.013	0.795	**−0.147**	**0.001**	0.006	0.884	**−0.092**	**0.017**
**Memory** *	−0.037	0.532	**−0.206**	**2.66E‐04**	−0.012	0.822	**−0.150**	**0.003**
**Visuospatial function**	−0.041	0.471	−0.084	0.095	−0.009	0.854	−0.031	0.493

*Note*: Model 1 adjusted for age, state of residence, and top 10 genetic PCs.

Model 2 adjusted for age, state of residence, top 10 genetic PCs, education (less than lower secondary education, upper secondary or vocational training and tertiary education), literacy, urban/rural residence, caste, and quintiles of per capita household consumption.

Beta coefficient and *p*‐value in bold indicates statistically significant association at *p* < 0.05.

Asterisk (*) denotes cognitive measures for which a statistically significant interaction term was observed.

Abrreviations: *APOE*, apolipoprotein E; HMSE, Hindi mental state examination; PCs, principal components.

Table S8 provides the results from testing the *APOE* ε4 by education interaction terms. Using the three‐level education variable, all interaction terms between *APOE* ε4 and education were not statistically significant except the interaction between ε4 and upper secondary or vocational training on visuospatial function. A closer examination of the education‐stratified analysis showed that most significant ε4 associations were predominantly observed in the less than lower secondary education group (Table S9). Given that this group constituted ∼75% of our sample, with more than 64% of them having no formal education, we further explored the *APOE* ε4 by education interaction by separating our sample into two categories: those with no formal education (0 years of education) and those with any type of formal education (> 0 years of education). We found no interaction between *APOE* ε4 and the dichotomous education variable on any of the cognitive measures (Table S10). However, when we examined the ε4 associations with cognitive measures separately within these two subgroups, we observed that ε4 was associated with all cognitive measures except visuospatial function among individuals without formal education in Model 1 (Table S11). In contrast, among those who had received some education, ε4 was only associated with HMSE score and orientation domain score. This pattern of association largely persisted in Model 2, except for the loss of significance in the associations between ε4 and executive function and language/fluency among those with no formal education.

### 3.4 Sensitivity analysis

Sensitivity analyses examining *APOE* genotypes showed that the main effect of ε3/ε4 compared to ε3/ε3, as well as its potential modification by age, sex, and education closely mirrored what we observed for ε4 carriers in the primary analyses (Tables S12‐S17). Furthermore, even though not statistically significant, we found that the effect of ε4/ε4 on cognitive function was in the expected direction, with lower scores observed among ε4/ε4 compared to ε3/ε3 in the full sample and within sex and education subgroups. Contrary to our expectation based on previous literature, we did not consistently observe a clear dose‐response relationship between the number of ε4 alleles and worse cognitive function, as indicated by the smaller magnitude of effect in ε4/ε4 homozygotes compared to ε3/ε4 heterozygotes in the full sample, as well as within subgroups of male sex and 0 years of education (Tables S12, S15, and S17). However, we caution that our sample included only a small number of individuals carrying the ε4/ε4 genotype (*N* = 36), which likely contributed to our inability to establish statistically significant associations or interactions. Finally, results from the sensitivity analyses also revealed an association between *APOE* ε2/ε3 genotype and lower visuospatial function when compared to ε3/ε3 (Table S12). We did not detect any significant interactions between the ε2/ε3 genotype and age, sex, or education (Tables S13, S14, and S16). Nevertheless, an adverse effect of the ε2/ε3 genotype on visuospatial function was found among females but not among males (Table S15).

Results from sensitivity analyses further controlling for vascular risk factors also yielded similar findings to our main analyses (Tables S18 and S19). The main effect of *APOE* ε4 and its interactions with age were negligibly different from those observed in the main analyses. Interactions between *APOE* ε4 and sex on language/fluency and memory were slightly attenuated but remained in the same direction and marginally significant (*p* = 0.062 for language/fluency and *p* = 0.052 for memory).

## 4 DISCUSSION

In this large, nationally representative sample of Indians aged 60 and older, *APOE* ε4 carrier status was associated with lower cognitive function, both globally and in multiple specific domains. We also found that females may be more susceptible to ε4 effects on memory and language/fluency, while older individuals may be more susceptible to ε4 effects on HMSE score, orientation, and language/fluency. The ε4 frequency was 10.8% in our sample, which is consistent with the 10.0% ε4 frequency reported in the Genome Aggregation Database (gnomAD) for South Asians^51^ and reaffirms the relatively lower ε4 prevalence among South Asians compared to the global frequency of 14%.^3^

We found modest adverse effects of *APOE* ε4 on a broad range of cognitive domains among older Indians. This finding contributes to the growing body of literature indicating that ε4 effects on cognitive function vary across racial and ethnic groups. For example, a previous study found that ε4 was associated with cognitive decline in middle‐aged Whites but not African Americans.^32^ In Hispanic and Asian populations, evidence of the ε4 effect on cognition is inconsistent, with some studies reporting a lack of association.^52^, ^53^ Direct comparison of ε4 effects on cognition is challenging as methodological factors, including measurement and modeling of cognitive measures, may vary substantially across studies. Nonetheless, the observed domain specificity of ε4 effects on cognition in our study is in line with two meta‐analyses of cognitively healthy European ancestry individuals which indicate that ε4 carriers perform worse on global cognition and episodic memory measures, but not on language/fluency (verbal ability) and visuospatial function.^22^, ^54^ In contrast, we did not find an association between ε4 carrier status and executive function after adjusting for sociodemographic and socioeconomic factors. These results also differ slightly from earlier LASI‐DAD studies in 932 participants,^55^, ^56^ which found no significant ε4 effects on cognition, potentially due to inadequate power and a more restricted geographic sample.

We also found a relatively consistent, though not always statistically significant, pattern across cognitive measures whereby the magnitudes of the ε4 effects on cognition appeared stronger among older individuals. This is in line with our hypothesis and several studies indicating that the negative effect of ε4 on cognitive function increases with advancing age.^22^, ^57^, ^58^ The molecular mechanisms underlying the potential age‐dependent ε4 effect on cognitive function remain unknown. One possibility is the resource modulation hypothesis,^58^ which posits that effects of common genetic variants, such as *APOE*, may become stronger as neurochemical and anatomical brain resources decline with age.^59^, ^60^ Conversely, the observed stronger association between ε4 and cognition with advancing age might reflect the increased presence of dementia cases in older individuals, as ε4 is more robustly associated with dementia. However, not all studies have observed this interaction.^54^, ^61^ In fact, one study showed that ε4 effects on global cognitive functioning and episodic memory decreased with age, although these findings were not statistically significant.^54^ These inconsistencies across studies may be attributed to methodologic differences, such as sample size, age range of the participants, and measurements of cognitive function.

We found that the ε4 association on cognition was stronger in females than males for language/fluency and memory, which is consistent with our hypothesis and a growing body of literature. Earlier research indicates that the ε4 allele confers a greater risk for AD in women than men,^13^, ^62^ with recent studies confirming its more pronounced effect on AD biomarkers and cognitive function in females.^18^, ^63^, ^64^ Although the precise biological mechanisms underlying these observed sex differences remain largely unclear, there is compelling biological plausibility. For example, *APOE* may interact with estrogen through various pathways, exacerbating the adverse effects of estrogen fluctuations/loss during perimenopause and postmenopause on cognitive function and AD.^65^ Alternatively, the observed sex differences in our sample may be partly due to a more pronounced selection bias among males. *APOE* ε4 has pleiotropic effects on cardiovascular disease and mortality, which are also associated with being male.^19^ Given the older age of our LASI‐DAD sample, it is likely to include healthier male ε4 carriers who demonstrate resilience against the detrimental ε4 effects on cognitive function and age‐related diseases. Consequently, our study may underestimate the association between ε4 and cognition in males. Indeed, while our findings align with most studies examining sex differences, a few studies, including a recent analysis of UK Biobank participants with white British ancestry, found no interactions between ε4 and sex on cognitive abilities.^66^ Further investigation is required to elucidate the observed sex‐specific ε4 effects on cognitive function and dementia in diverse populations.

In our study, higher education was associated with better cognitive performance as expected. However, contrary to our hypothesis, the *APOE* ε4 association with cognitive function did not differ significantly across education groups. Previous studies in European ancestry demonstrated that higher education may counteract the negative effects of ε4 on cognitive function and dementia by providing greater cognitive reserve, which enables individuals to better cope with brain pathologies, thereby mitigating genetic risk associated with ε4.^16^, ^67^ While we found no evidence of interaction when including a cross‐product interaction term between *APOE* ε4 and > 0 years of education, education‐stratified analysis showed that the magnitude of the association between ε4 and most cognitive measures trended greater in those who never attended school compared to those who did. The lack of interactions may partially stem from analyzing education as a dichotomous variable based on having ever attended school, as some studies suggest that the interaction between ε4 and education may be at the tertiary level.^67^ Although we examined and found no interaction at the tertiary level, we may have lacked statistical power to detect such an interaction given that we had only 95 participants with tertiary education. Further, the moderate overall sample size may have limited our power to detect smaller effects. The complex social hierarchy and high social stratification in India, which we could not completely capture, might also influence this interaction.

Sensitivity analyses using *APOE* genotypes further revealed that ε2/ε3 was associated with lower visuospatial function compared to ε3/ε3. While the ε2 allele is generally considered protective against AD, previous studies of cognition have yielded inconsistent findings. For instance, one study found worse visuospatial attention in mid‐adulthood ε2 carriers,^68^ whereas another study in older Koreans without dementia reported better visuospatial performance in ε2 carriers.^69^ Additional research is needed to replicate our findings and elucidate the ε2 effect on cognitive function.

There is a higher prevalence of cardiometabolic risk factors in LASI‐DAD compared to nationally representative US‐based aging studies.^70^ However, most traditional cardiovascular risk factors were not associated with cognitive function in LASI‐DAD.^71^ In our study, adjusting for vascular factors did not alter the main findings, except for the interactions between ε4 and sex on language/fluency and memory, which remained directionally consistent but only marginally significant. While this may be due to reduced power from the smaller number of participants who had data on vascular risk factors, it is possible that the interaction between ε4 and sex on cognition may be confounded or moderated by these vascular factors. Future research is warranted to elucidate the role of vascular risk factors in ε4 associations with cognitive function, particularly in South Asians.

This study has limitations. First, we were unable to reliably estimate the dosage effect of ε4 due to the rarity of ε4 homozygotes (*N* = 36). Second, LASI‐DAD only included participants aged 60 or older, which may have induced selection bias. Our results might underestimate the true association between ε4 and cognition since older adults carrying ε4 and with poorer cognitive function might be underrepresented due to morbidity or death. Contrary to previous studies that reported a decrease in ε4 frequency with increasing age,^72^, ^73^ our study showed no significant age differences in ε4 frequency. We did observe a decreasing trend in ε4 frequency up to age 85, followed by an increase, which may be the result of underlying population structure differences or the small sample size of the oldest age groups. Third, despite adjusting for many potential confounders, there may be residual confounding from lifestyle factors and environmental toxicants. Fourth, all socioeconomic characteristics were self‐reported and may be subject to response bias. Fifth, we were unable to examine longitudinal ε4 effects due to the cross‐sectional study design. Future studies using longitudinal cognitive measures in LASI‐DAD, which are currently being collected, could provide further insights into the interplay among ε4, sociodemographic, and cognitive aging in South Asians. Finally, our findings need to be replicated in other South Asian/Indian samples.

In conclusion, this is the first study to investigate the effect of *APOE* ε4 and its potential modification by sociodemographic factors on cognitive function in a nationally representative sample of older Indians. Our study provides important initial insights into the relationship between the strongest known genetic risk factor for AD and cognitive function in the South Asians population. Future research is warranted to provide a better understanding of the genetic influences on cognition and dementia within this genetically and socially diverse population.

## CONFLICT OF INTEREST STATEMENT

The authors have no conflicts of interest to disclose. Author disclosures are available in the Supporting information.

## CONSENT STATEMENT

Informed consent was obtained from all participants included in the study.

## Supporting information

Supporting information

Supporting information

## Data Availability

WGS data for LASI‐DAD are available from the National Institute on Aging Genetics of Alzheimer's Disease Data Storage Site (NIAGADS), accession number: NG00067–ADSP Umbrella. Phenotype data is available at the Gateway to Global Aging website, https://g2aging.org/.
