# Supplementary material for: Effect of apolipoprotein E ε4 and its modification by sociodemographic characteristics on cognitive measures in South Asians from LASI‐DAD
Source: Alzheimers Dement. 2024 Jun 18;20(7):4854–67. doi: 10.1002/alz.14052 (PMC11247697; doi:10.1002/alz.14052)
Supplement: Supplementary file 1 — Supporting information [file ALZ-20-4854-s002.docx]

**Supplementary Materials**

Table of Contents

[Supplementary Figures 3](#_Toc164851031)

[Supplementary Figure 1. Study flow chart. 3](#_Toc164851032)

[Supplementary Figure 2. Allele frequency of *APOE* ε2 and ε4 by 5-year age group. 4](#_Toc164851033)

[Supplementary Figure 3. Associations of *APOE* ε4 carrier status with cognitive measures across 5-year age groups. 5](#_Toc164851034)

[Supplementary Tables 6](#_Toc164851035)

[Supplementary Table 1. Cognitive measures across LASI-DAD participants with and without whole genome sequencing. 6](#_Toc164851036)

[Supplementary Table 2. Tests included in the cognitive battery by domain. 7](#_Toc164851037)

[Supplementary Table 3. Allele frequencies of *APOE* ε2, ε3, and ε4 in the full study sample, and by 5-year age group ranging from 60 years to ≥85 years. 8](#_Toc164851038)

[Supplementary Table 4. Correlation among cognitive domain scores in LASI-DAD. 9](#_Toc164851039)

[Supplementary Table 5. Descriptive statistics of vascular risk factors by *APOE* ε4 carrier status among the analytic sample. 10](#_Toc164851040)

[Supplementary Table 6. Associations of *APOE* ε4 and sociodemographic characteristics with cognitive measures (Model 1). 11](#_Toc164851041)

[Supplementary Table 7. Associations of *APOE* ε4 and sociodemographic characteristics with cognitive measures (Model 2). 12](#_Toc164851042)

[Supplementary Table 8. Two-way interaction between *APOE* ε4 carrier and educational level on cognitive measures. 13](#_Toc164851043)

[Supplementary Table 9. Associations between *APOE* ε4 carrier and cognitive measures stratified by education level. 15](#_Toc164851044)

[Supplementary Table 10. Two-way interaction between *APOE* ε4 carrier and dichotomous education level on cognitive measures. 16](#_Toc164851045)

[Supplementary Table 11. Associations between *APOE* ε4 carrier and cognitive measures stratified by education (dichotomized at having received some formal schooling). 17](#_Toc164851046)

[Supplementary Table 12. Main effects of *APOE* genotypes on cognitive measures in LASI-DAD. 18](#_Toc164851047)

[Supplementary Table 13. Two-way interaction between *APOE* genotypes and continuous age on cognitive measures. 19](#_Toc164851048)

[Supplementary Table 14. Two-way interaction between *APOE* genotypes and sex on cognitive measures. 20](#_Toc164851049)

[Supplementary Table 15. Associations between *APOE* genotypes and cognitive measures stratified by sex. 21](#_Toc164851050)

[Supplementary Table 16. Two-way interaction between *APOE* genotypes and educational level (dichotomized at having received some formal schooling) on cognitive measures. 22](#_Toc164851051)

[Supplementary Table 17. Associations between *APOE* genotype and cognitive measures stratified by education level (dichotomized at having received some formal schooling). 23](#_Toc164851052)

[Supplementary Table 18. Significant associations between *APOE* ε4 carrier status and cognitive measures additionally adjusted for vascular risk factors in LASI-DAD. 24](#_Toc164851053)

[Supplementary Table 19. Significant *APOE* ε4 interactions after additionally adjusted for vascular risk factors in LASI-DAD. 25](#_Toc164851054)

# Supplementary Figures


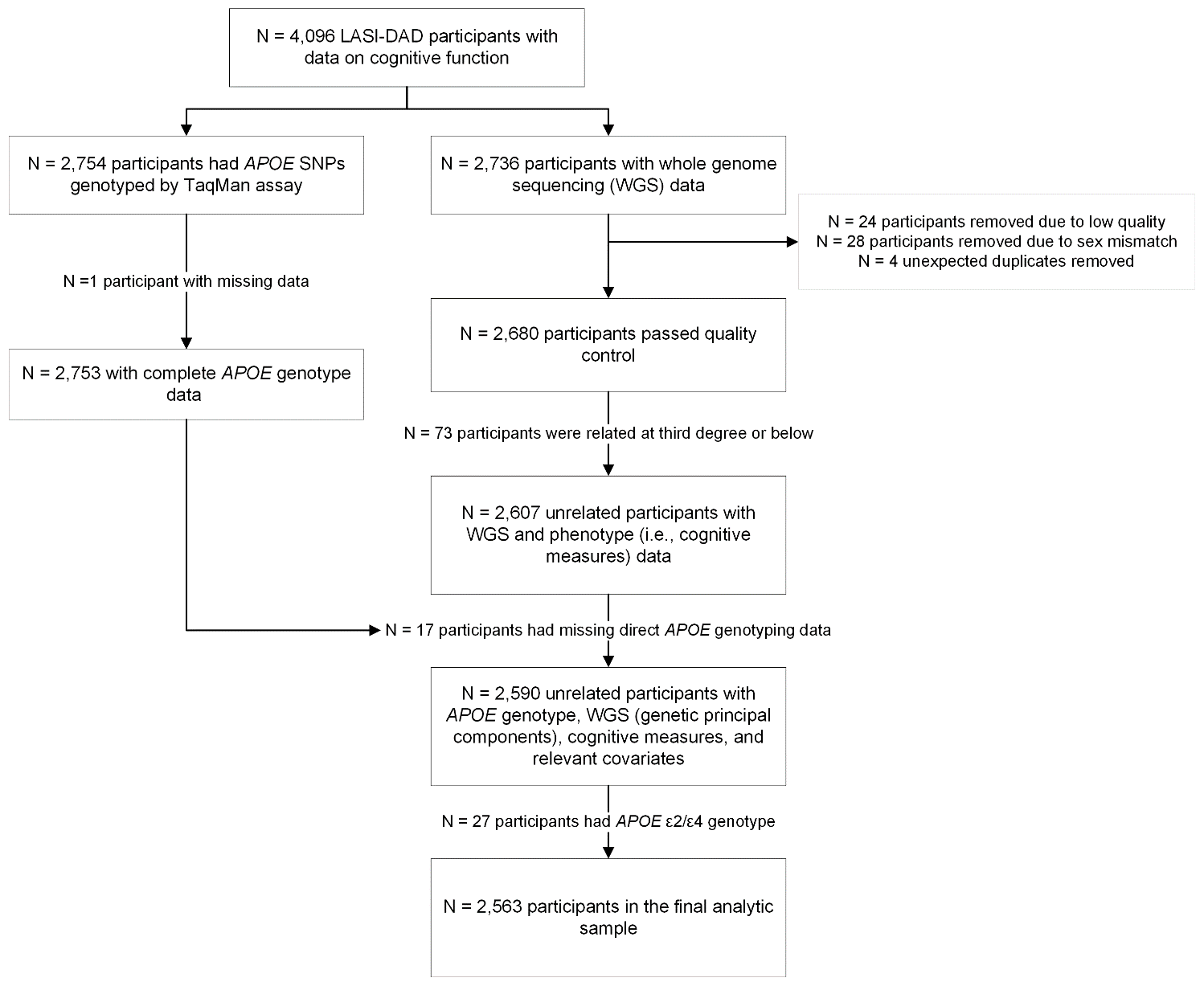


## Supplementary Figure 1. Study flow chart.


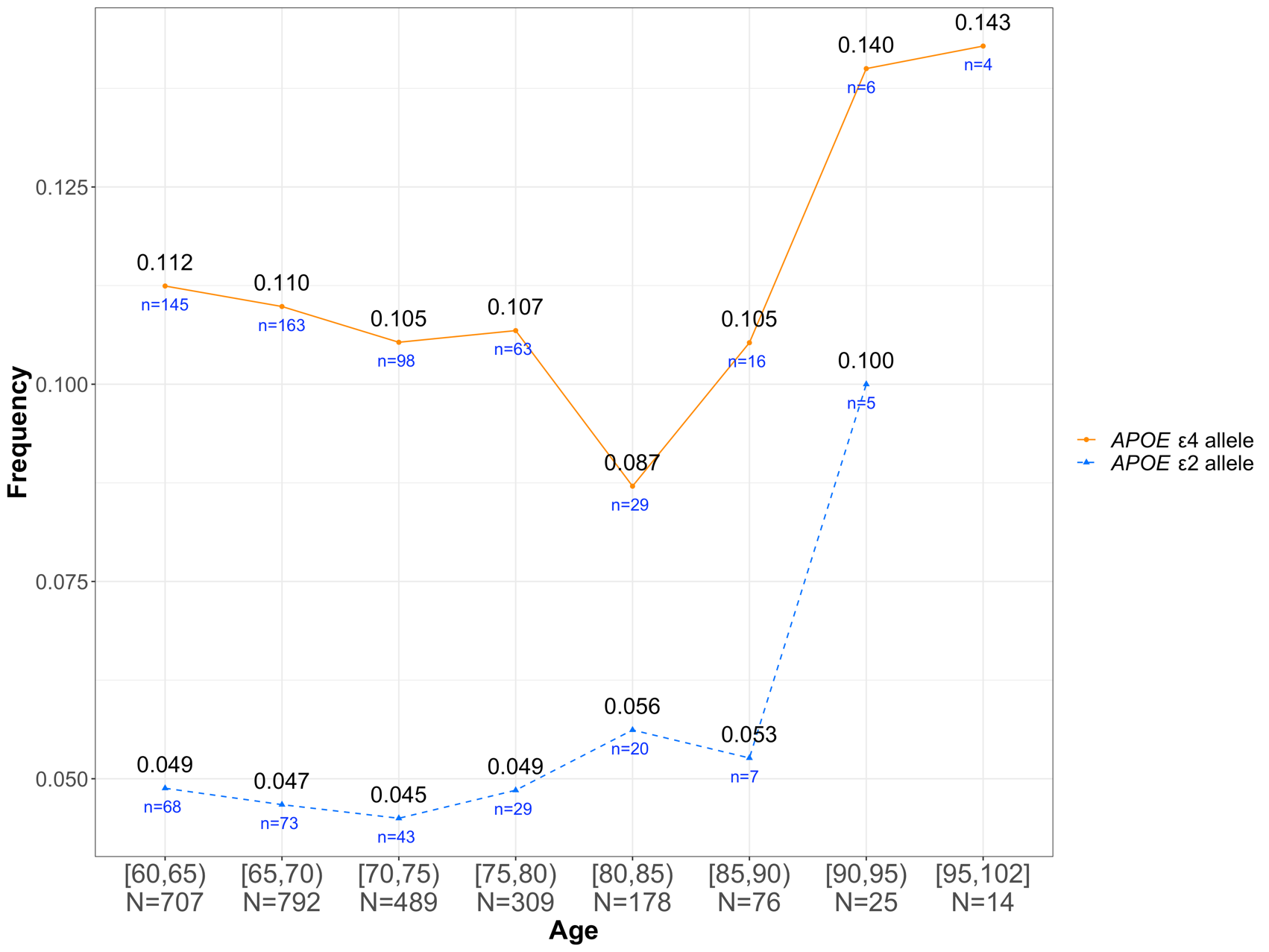


Supplementary Figure 2. Allele frequency of *APOE* ε2 and ε4 by 5-year age group.

*APOE* ε4 frequencies are indicated by orange dots and connected by solid orange lines, while *APOE* ε2 frequencies are indicated with blue triangles and connected by dashed blue lines. The numbers under each curve represent the number of participants carrying the respective allele within each age group.

## Supplementary Figure 3. Associations of *APOE* ε4 carrier status with cognitive measures across 5-year age groups.

Model adjusted for sex (male), state of residence, top 10 genetic PCs, education (upper secondary or vocational training and tertiary education), literacy, urban/rural residence, caste, and quintiles of per capita household consumption.

Grey shaded areas correspond to the 95% confidence interval of the ε4 effect estimate calculated from the stratified analysis by 5-year age group.

# Supplementary Tables

## Supplementary Table 1. Cognitive measures across LASI-DAD participants with and without whole genome sequencing.

| **Cognitive measures** | **Participants with**  **WGS data** | **Participants without WGS data** | ***P* value** |
| --- | --- | --- | --- |
|  | **(N = 2,736)** | **(N = 1,360)** |  |
| HMSE score, mean (SD) | 22.66 (5.4) | 22.45 (5.74) | 0.253 |
| General cognitive function, mean (SD) | 0.00 (0.92) | -0.04 (0.97) | 0.163 |
| Cognitive domain scores, mean (SD) |  |  | 0.407 |
| Orientation | -0.01 (0.90) | -0.03 (0.93) | 0.013 |
| Executive function | -0.03 (0.80) | -0.09 (0.85) | 0.357 |
| Language/fluency | -0.03 (0.80) | -0.01 (0.83) | 0.106 |
| Memory | 0.02 (0.94) | -0.04 (1.00) | 0.053 |
| Visuospatial function | 0.03 (0.83) | -0.02 (0.84) | 0.253 |

Abbreviations: LASI-DAD = Diagnostic Assessment of Dementia for the Longitudinal Aging Study of India; WGS = whole genome sequencing; SD = standard deviation. HMSE = Hindi Mental State Examination.

Note: p-value calculated from t-test.

## Supplementary Table 2. Tests included in the cognitive battery by domain.

| **General domain** | **Broad domains** | **Observed cognitive test items** |
| --- | --- | --- |
| General cognitive function | Orientation | Name the current month, year, season, day of the week, day of the month, state, city, hospital name (or district if at home), area of town/village or street name, and the Prime Minister. |
|  | Language/fluency | Animal naming, writing or saying a sentence, phrase repetition, naming of common objects by sight (watch, pencil), naming of common objects by description (elbow, hammer, scissors, coconut, window), following a read or acted command to close one's eyes, and completing a 3-stage task. |
|  | Memory | Immediate recall of a 10-word list, a three-word list, the Brave Man story learning test, and the Logical Memory test;  Delayed recall of a 10-word list, a three-world list, the Brave Man story learning test, the Logical Memory test, and the constructional praxis test;  Recognition recall of a 10-word list and the Logical Memory test. |
|  | Executive function | Abstract reasoning measured by Ravens progressive matrices task, clock drawing, two trials of the Go-No-Go test; Attention/speed measured by a numeracy task, backwards day counting, symbol cancellation, and the Digit Span forwards and backwards tasks. |
|  | Visuospatial function | Constructional praxis (drawing a circle, rectangle, cube, and diamond), and interlocking pentagons |

**Note:** Adapted from Gross AL et. al., *J Am Geriatr Soc* 2020;68:S11–9.

## Supplementary Table 3. Allele frequencies of *APOE* ε2, ε3, and ε4 in the full study sample, and by 5-year age group ranging from 60 years to ≥85 years.

|  | **Total sample (N = 2,590)** | **[60,65) (n = 707)** | **[65,70) (n = 792)** | **[70,75) (n =489)** | **[75,80) (n = 309)** | **[80,85) (n = 178)** | **≥85 (n = 115)** | **P value** |
| --- | --- | --- | --- | --- | --- | --- | --- | --- |
| **Allele frequencies** | | | | | | | |  |
| ε2 | 0.048 | 0.049 | 0.047 | 0.045 | 0.049 | 0.056 | 0.057 | 0.950^‡^ |
| ε3 | 0.844 | 0.839 | 0.843 | 0.850 | 0.845 | 0.857 | 0.826 | 0.909^‡^ |
| ε4 | 0.108 | 0.112 | 0.110 | 0.105 | 0.107 | 0.087 | 0.117 | 0.813^‡^ |
| ***APOE* genotype (n, %)** | | | | | | | | 0.904^†^ |
| ε2/ε2 | 5 (0.2%) | 1 (0.1%) | 1 (0.1%) | 1 (0.2%) | 1 (0.3%) | 0 | 1 (0.9%) |  |
| ε2/ε3 | 213 (8.2%) | 57 (8.1%) | 63 (8.0%) | 41 (8.4%) | 23 (7.4%) | 19 (10.7%) | 10 (8.7%) |  |
| ε2/ε4 | 27 (1.0%) | 10 (1.4%) | 9 (1.1%) | 1 (0.2%) | 5 (1.6%) | 1 (0.6%) | 1 (0.9%) |  |
| ε3/ε3 | 1848 (71.4%) | 504 (71.3%) | 565 (71.3%) | 349 (71.4%) | 222 (71.8%) | 130 (73.0%) | 78 (67.8%) |  |
| ε3/ε4 | 461 (17.8%) | 121 (17.1%) | 143 (18.1%) | 92 (18.8%) | 55 (17.8%) | 26 (14.6%) | 24 (20.9%) |  |
| ε4/ε4 | 36 (1.4%) | 14 (2.0%) | 11 (1.4%) | 5 (1.0%) | 3 (1.0%) | 2 (1.1%) | 1 (0.9%) |  |
| ***APOE* ε4 carrier status (n, %)** | | | | | | | | 0.817^†^ |
| ε4 carrier | 524 (20.2%) | 145 (20.5%) | 163 (20.6%) | 98 (20.0%) | 63 (20.4%) | 29 (16.3%) | 26 (22.6%) |  |
| ε4 noncarrier | 2,066 (79.8%) | 562 (79.5%) | 629 (79.4%) | 391 (80.0%) | 246 (79.6%) | 149 (83.7%) | 89 (77.4%) |  |
| ***APOE* ε4 carrier status after removing the *APOE* ε2/ε4 genotype (n, %)** | | | | | | | | 0.843^†^ |
| ε4 carrier | 497 (19.2%) | 135 (19.1%) | 154 (19.4%) | 97 (19.8%) | 58 (18.8%) | 28 (15.7%) | 25 (21.7%) |  |
| ε4 noncarrier | 2,066 (79.8%) | 562 (79.5%) | 629 (79.4%) | 391 (80.0%) | 246 (79.6%) | 149 (83.7%) | 89 (77.4%) |  |

Abbreviations: APOE = apolipoprotein E; LASI-DAD = Diagnostic Assessment of Dementia for the Longitudinal Aging Study of India; HMSE = Hindi Mental State Examination.

*Individuals with the *APOE* ε2/ε4 genotype were not included.

^†^ p-value calculated from chi-square test across the 5-year age groups.

^‡^ p-value calculated from chi-square test for equality of proportions among the 5-year age groups.

## Supplementary Table 4. Correlation among cognitive domain scores in LASI-DAD.

|  | HMSE score | General cognitive function | Executive function | Orientation | Language/ fluency | Memory | Visuospatial function |
| --- | --- | --- | --- | --- | --- | --- | --- |
| HMSE score | 1.000 |  |  |  |  |  |  |
| General cognitive function | 0.867 | 1.000 |  |  |  |  |  |
| Executive function | 0.789 | 0.948 | 1.000 |  |  |  |  |
| Orientation | 0.856 | 0.841 | 0.733 | 1.000 |  |  |  |
| Language fluency | 0.773 | 0.816 | 0.713 | 0.655 | 1.000 |  |  |
| Memory | 0.667 | 0.803 | 0.684 | 0.610 | 0.632 | 1.000 |  |
| Visuospatial function | 0.560 | 0.733 | 0.673 | 0.542 | 0.491 | 0.493 | 1.000 |

Abbreviations: LASI-DAD, Longitudinal Aging Study in India-Diagnostic Assessment of Dementia; HMSE = Hindi Mental State Examination.

## Supplementary Table 5. Descriptive statistics of vascular risk factors by *APOE* ε4 carrier status among the analytic sample.

|  | **Total** | ***APOE* ε4 noncarrier** | ***APOE* ε4 carrier** | **P value^a^** |
| --- | --- | --- | --- | --- |
| High blood pressure (n = 2,554) | 1024 (40.0%) | 839 (40.6%) | 185 (37.2%) | 0.195 |
| Smoking (ever smoked, n = 2,547) | 566 (22.1%) | 457 (22.1%) | 109 (21.9%) | 0.989 |
| Diabetes (n = 2,554) | 462 (18.0%) | 374 (18.1%) | 88 (17.7%) | 0.878 |
| High cholesterol (n = 2,554) | 133 (5.2%) | 110 (5.3%) | 23 (4.6%) | 0.546 |
| Body mass index (n = 2,364) |  |  |  | 0.001 |
| Underweight | 486 (19.0%) | 367 (17.8%) | 119 (23.9%) |  |
| Normal weight | 1212 (47.3%) | 986 (47.7%) | 226 (45.5%) |  |
| Overweight/obese | 666 (26.0%) | 560 (27.1%) | 106 (21.3%) |  |

Abbreviations: APOE = apolipoprotein E; LASI-DAD = Diagnostic Assessment of Dementia for the Longitudinal Aging Study of India; SD = standard deviation. HMSE = Hindi Mental State Examination.

Note: Individuals with the *APOE* ε2/ε4 genotype were excluded from the sample. N (%) are reported unless otherwise specified.

^a^ p-value calculated from Chi-square test.

## Supplementary Table 6. Associations of *APOE* ε4 and sociodemographic characteristics with cognitive measures (Model 1).

| **Model 1 (n = 2,563)** | **HMSE score** | | **General cognitive function** | | **Executive function** | | **Orientation** | | **Language/fluency** | | **Memory** | | **Visuospatial function** | |
| --- | --- | --- | --- | --- | --- | --- | --- | --- | --- | --- | --- | --- | --- | --- |
|  | **Beta** | **P value** | **Beta** | **P value** | **Beta** | **P value** | **Beta** | **P value** | **Beta** | **P value** | **Beta** | **P value** | **Beta** | **P value** |
| *APOE* ε4 | -0.978 | 2.44E-05 | -0.142 | 1.69E-04 | -0.117 | 0.002 | -0.141 | 2.86E-05 | -0.095 | 0.005 | -0.134 | 0.001 | -0.063 | 0.097 |
| Age | -0.184 | 1.39E-46 | -0.034 | 1.22E-58 | -0.030 | 9.93E-48 | -0.023 | 1.45E-34 | -0.022 | 4.73E-32 | -0.036 | 7.72E-57 | -0.023 | 9.13E-28 |
| Male | 3.342 | 1.30E-69 | 0.634 | 1.16E-91 | 0.641 | 1.33E-94 | 0.654 | 1.54E-118 | 0.384 | 1.01E-44 | 0.208 | 1.94E-10 | 0.452 | 6.52E-49 |
| Bihar | 3.024 | 1.91E-05 | 0.562 | 1.09E-06 | 0.751 | 6.35E-11 | 0.347 | 0.001 | 0.273 | 0.008 | 0.065 | 0.601 | 0.521 | 6.80E-06 |
| Assam | 3.809 | 4.87E-05 | 0.930 | 1.29E-09 | 1.045 | 7.16E-12 | 0.404 | 0.003 | 0.906 | 4.22E-11 | 0.066 | 0.690 | 0.971 | 2.70E-10 |
| West Bengal | 4.445 | 1.82E-10 | 0.934 | 2.51E-16 | 1.120 | 6.62E-23 | 0.368 | 2.76E-04 | 0.810 | 2.19E-15 | 0.507 | 3.91E-05 | 0.669 | 4.54E-09 |
| Orissa | 1.820 | 0.009 | 0.697 | 1.21E-09 | 0.896 | 4.27E-15 | 0.267 | 0.009 | 0.182 | 0.076 | 0.134 | 0.280 | 0.950 | 1.92E-16 |
| Madhya Pradesh | 2.097 | 0.007 | 0.332 | 0.008 | 0.573 | 4.69E-06 | 0.237 | 0.035 | 0.066 | 0.556 | -0.352 | 0.010 | 0.472 | 1.87E-04 |
| Gujarat | 3.334 | 3.13E-06 | 0.885 | 3.85E-14 | 0.995 | 1.29E-17 | 0.551 | 1.28E-07 | 0.555 | 1.13E-07 | 0.200 | 0.113 | 0.916 | 6.45E-15 |
| Maharashtra | 6.490 | 7.21E-19 | 1.480 | 7.12E-35 | 1.705 | 8.53E-46 | 0.841 | 2.65E-15 | 0.782 | 2.27E-13 | 0.783 | 1.35E-09 | 1.226 | 1.75E-24 |
| Karnataka | 6.346 | 5.69E-13 | 1.358 | 4.24E-21 | 1.578 | 4.55E-28 | 0.598 | 2.91E-06 | 1.122 | 3.32E-18 | 0.794 | 3.42E-07 | 0.949 | 4.44E-11 |
| Punjab | 5.034 | 1.25E-12 | 0.879 | 2.95E-14 | 0.883 | 1.60E-14 | 0.670 | 8.54E-11 | 0.645 | 4.57E-10 | 0.557 | 8.85E-06 | 0.678 | 4.83E-09 |
| Kerala | 8.726 | 2.50E-22 | 1.829 | 2.02E-35 | 1.897 | 2.56E-38 | 1.010 | 9.44E-15 | 1.462 | 1.07E-28 | 1.297 | 3.03E-16 | 1.176 | 1.01E-15 |
| Tamil Nadu | 7.068 | 3.12E-15 | 1.601 | 1.22E-27 | 1.781 | 5.51E-34 | 0.778 | 2.33E-09 | 1.338 | 2.67E-24 | 0.968 | 1.02E-09 | 1.130 | 1.33E-14 |
| Telangana | 3.480 | 1.56E-05 | 0.999 | 3.60E-14 | 1.157 | 1.19E-18 | 0.364 | 0.002 | 0.702 | 2.67E-09 | 0.488 | 0.001 | 1.012 | 2.08E-14 |
| Uttranchal | 2.339 | 0.003 | 0.548 | 2.72E-05 | 0.696 | 8.53E-08 | 0.318 | 0.006 | 0.343 | 0.003 | -0.045 | 0.748 | 0.595 | 5.71E-06 |
| Haryana | 1.873 | 0.003 | 0.326 | 0.002 | 0.522 | 4.28E-07 | 0.209 | 0.024 | 0.132 | 0.156 | -0.195 | 0.084 | 0.344 | 0.001 |
| Delhi | 3.093 | 5.29E-06 | 0.820 | 1.63E-13 | 1.042 | 5.21E-21 | 0.544 | 4.06E-08 | 0.458 | 3.90E-06 | 0.246 | 0.041 | 0.508 | 4.99E-06 |
| Rajasthan | 2.205 | 0.001 | 0.400 | 2.62E-04 | 0.646 | 3.28E-09 | 0.192 | 0.050 | 0.453 | 4.15E-06 | -0.397 | 0.001 | 0.296 | 0.007 |
| Uttar Pradesh | 3.388 | 2.73E-07 | 0.532 | 7.30E-07 | 0.719 | 1.80E-11 | 0.433 | 6.23E-06 | 0.355 | 2.25E-04 | -0.162 | 0.164 | 0.516 | 1.71E-06 |
| PC1 | 67.706 | 2.59E-18 | 14.069 | 1.71E-28 | 13.618 | 3.90E-27 | 9.039 | 1.16E-15 | 9.919 | 2.14E-18 | 12.653 | 3.72E-20 | 8.605 | 1.06E-11 |
| PC2 | 29.839 | 0.001 | 7.682 | 5.42E-07 | 8.013 | 1.46E-07 | 4.169 | 0.002 | 3.531 | 0.010 | 6.819 | 4.22E-05 | 6.589 | 1.83E-05 |
| PC3 | 12.212 | 0.155 | 2.670 | 0.057 | 2.470 | 0.076 | 1.528 | 0.222 | 0.704 | 0.575 | 2.860 | 0.060 | 3.004 | 0.033 |
| PC4 | 27.901 | 1.71E-05 | 5.421 | 3.08E-07 | 4.967 | 2.35E-06 | 3.485 | 2.26E-04 | 2.411 | 0.011 | 6.086 | 1.25E-07 | 4.531 | 2.00E-05 |
| PC5 | 16.919 | 0.001 | 3.779 | 1.25E-05 | 4.163 | 1.30E-06 | 2.390 | 0.002 | 1.090 | 0.159 | 3.244 | 0.001 | 3.339 | 1.20E-04 |
| PC6 | 5.613 | 0.281 | 0.453 | 0.594 | 0.061 | 0.942 | 0.697 | 0.358 | 1.426 | 0.061 | 0.072 | 0.938 | -0.534 | 0.531 |
| PC7 | 4.376 | 0.362 | 0.770 | 0.325 | 1.312 | 0.092 | 0.321 | 0.646 | 0.104 | 0.882 | 0.541 | 0.525 | 0.385 | 0.624 |
| PC8 | -5.569 | 0.286 | -0.736 | 0.388 | -1.261 | 0.137 | -0.470 | 0.537 | -0.341 | 0.655 | 0.594 | 0.521 | -0.589 | 0.491 |
| PC9 | -10.755 | 0.070 | -1.983 | 0.040 | -1.543 | 0.109 | -2.224 | 0.010 | -1.988 | 0.022 | -1.001 | 0.341 | -1.021 | 0.293 |
| PC10 | 1.528 | 0.760 | 0.073 | 0.929 | 0.379 | 0.640 | -0.125 | 0.863 | 0.080 | 0.913 | 0.239 | 0.787 | -0.562 | 0.492 |
| **Total R^2^** | **0.281** | | **0.339** | | **0.328** | | **0.299** | | **0.307** | | **0.252** | | **0.186** | |

Abbreviations: APOE = apolipoprotein E; PC = principal component; HMSE = Hindi Mental State Examination.

## Supplementary Table 7. Associations of *APOE* ε4 and sociodemographic characteristics with cognitive measures (Model 2).

| **Model 2 (n = 2,548)** | **HMSE score** | | **General cognitive function** | | **Executive function** | | **Orientation** | | **Language/ fluency** | | **Memory** | | **Visuospatial function** | |
| --- | --- | --- | --- | --- | --- | --- | --- | --- | --- | --- | --- | --- | --- | --- |
|  | **Beta** | **P value** | **Beta** | **P value** | **Beta** | **P value** | **Beta** | **P value** | **Beta** | **P value** | **Beta** | **P value** | **Beta** | **P value** |
| *APOE* ε4 | -0.710 | 0.001 | -0.079 | 0.006 | -0.057 | 0.054 | -0.096 | 0.001 | -0.052 | 0.064 | -0.087 | 0.019 | -0.019 | 0.576 |
| Age | -0.154 | 1.98E-39 | -0.026 | 4.47E-58 | -0.022 | 3.18E-41 | -0.018 | 5.50E-28 | -0.017 | 4.00E-26 | -0.031 | 6.72E-49 | -0.016 | 7.29E-19 |
| Male | 1.813 | 2.91E-23 | 0.249 | 2.11E-23 | 0.286 | 4.05E-28 | 0.398 | 2.62E-53 | 0.092 | 1.62E-04 | -0.068 | 0.034 | 0.154 | 8.65E-08 |
| Bihar | 2.096 | 0.001 | 0.305 | 0.001 | 0.523 | 2.23E-08 | 0.180 | 0.050 | 0.033 | 0.704 | -0.103 | 0.372 | 0.330 | 0.001 |
| Assam | 1.008 | 0.247 | 0.290 | 0.015 | 0.462 | 1.97E-04 | -0.066 | 0.586 | 0.352 | 0.003 | -0.332 | 0.031 | 0.532 | 1.21E-04 |
| West Bengal | 1.691 | 0.009 | 0.287 | 0.001 | 0.519 | 2.35E-08 | -0.092 | 0.315 | 0.288 | 0.001 | 0.054 | 0.640 | 0.244 | 0.018 |
| Orissa | -1.026 | 0.119 | 0.031 | 0.730 | 0.292 | 0.002 | -0.216 | 0.019 | -0.390 | 1.10E-05 | -0.316 | 0.007 | 0.501 | 1.77E-06 |
| Madhya Pradesh | 1.344 | 0.058 | 0.123 | 0.206 | 0.385 | 1.38E-04 | 0.101 | 0.310 | -0.114 | 0.232 | -0.505 | 5.64E-05 | 0.331 | 0.003 |
| Gujarat | 0.785 | 0.235 | 0.252 | 0.005 | 0.416 | 1.00E-05 | 0.102 | 0.273 | 0.057 | 0.520 | -0.263 | 0.024 | 0.465 | 1.00E-05 |
| Maharashtra | 3.202 | 2.97E-06 | 0.660 | 2.40E-12 | 0.934 | 1.83E-21 | 0.276 | 0.004 | 0.176 | 0.056 | 0.180 | 0.135 | 0.633 | 6.42E-09 |
| Karnataka | 3.388 | 3.04E-05 | 0.647 | 6.44E-09 | 0.912 | 4.08E-15 | 0.097 | 0.394 | 0.594 | 5.99E-08 | 0.268 | 0.061 | 0.450 | 4.81E-04 |
| Punjab | 4.009 | 7.28E-10 | 0.589 | 3.96E-11 | 0.626 | 1.39E-11 | 0.481 | 1.31E-07 | 0.406 | 3.41E-06 | 0.343 | 0.003 | 0.453 | 1.14E-05 |
| Kerala | 3.894 | 3.16E-06 | 0.654 | 1.14E-08 | 0.799 | 2.07E-11 | 0.195 | 0.095 | 0.580 | 2.64E-07 | 0.442 | 0.003 | 0.331 | 0.012 |
| Tamil Nadu | 3.672 | 1.05E-05 | 0.751 | 5.15E-11 | 0.982 | 1.69E-16 | 0.198 | 0.090 | 0.695 | 6.36E-10 | 0.341 | 0.020 | 0.540 | 4.50E-05 |
| Telangana | 0.948 | 0.203 | 0.375 | 2.39E-04 | 0.574 | 6.46E-08 | -0.069 | 0.508 | 0.231 | 0.021 | 0.022 | 0.866 | 0.586 | 7.64E-07 |
| Uttranchal | 0.809 | 0.269 | 0.159 | 0.113 | 0.347 | 8.66E-04 | 0.052 | 0.609 | 0.011 | 0.914 | -0.318 | 0.014 | 0.323 | 0.005 |
| Haryana | 1.026 | 0.078 | 0.091 | 0.256 | 0.310 | 1.86E-04 | 0.054 | 0.511 | -0.060 | 0.444 | -0.365 | 3.94E-04 | 0.166 | 0.073 |
| Delhi | 0.830 | 0.199 | 0.224 | 0.011 | 0.477 | 2.31E-07 | 0.142 | 0.118 | 0.018 | 0.832 | -0.196 | 0.086 | 0.096 | 0.351 |
| Rajasthan | 1.235 | 0.047 | 0.125 | 0.142 | 0.407 | 4.25E-06 | 0.008 | 0.925 | 0.224 | 0.008 | -0.599 | 5.18E-08 | 0.075 | 0.450 |
| Uttar Pradesh | 2.046 | 0.001 | 0.145 | 0.084 | 0.351 | 5.85E-05 | 0.194 | 0.024 | 0.074 | 0.369 | -0.453 | 2.95E-05 | 0.231 | 0.018 |
| PC1 | 17.924 | 0.026 | 3.970 | 3.26E-04 | 4.007 | 4.77E-04 | 1.118 | 0.322 | 2.134 | 0.049 | 5.777 | 4.96E-05 | 2.491 | 0.052 |
| PC2 | 13.650 | 0.117 | 3.614 | 0.002 | 4.045 | 0.001 | 1.495 | 0.220 | 1.219 | 0.299 | 3.462 | 0.024 | 3.253 | 0.019 |
| PC3 | 13.905 | 0.083 | 2.722 | 0.013 | 2.473 | 0.030 | 1.797 | 0.110 | 1.094 | 0.312 | 2.613 | 0.065 | 2.878 | 0.024 |
| PC4 | 10.959 | 0.074 | 1.903 | 0.023 | 1.576 | 0.071 | 0.687 | 0.424 | 0.006 | 0.994 | 3.544 | 0.001 | 2.249 | 0.021 |
| PC5 | 10.217 | 0.036 | 2.247 | 0.001 | 2.638 | 1.44E-04 | 1.299 | 0.057 | -0.046 | 0.944 | 2.154 | 0.012 | 2.467 | 0.001 |
| PC6 | 1.948 | 0.681 | -0.250 | 0.700 | -0.608 | 0.367 | 0.113 | 0.865 | 0.927 | 0.146 | -0.473 | 0.572 | -0.879 | 0.242 |
| PC7 | 3.106 | 0.476 | 0.310 | 0.604 | 0.874 | 0.159 | 0.075 | 0.902 | -0.184 | 0.754 | 0.107 | 0.889 | 0.062 | 0.928 |
| PC8 | -1.382 | 0.771 | 0.135 | 0.835 | -0.469 | 0.487 | 0.268 | 0.687 | 0.449 | 0.482 | 1.271 | 0.129 | -0.293 | 0.697 |
| PC9 | -1.752 | 0.746 | 0.187 | 0.801 | 0.382 | 0.620 | -0.648 | 0.393 | -0.277 | 0.704 | 0.681 | 0.477 | 0.566 | 0.510 |
| PC10 | 1.051 | 0.816 | 0.117 | 0.849 | 0.429 | 0.504 | -0.207 | 0.743 | 0.055 | 0.928 | 0.297 | 0.709 | -0.420 | 0.558 |
| Education level |  |  |  |  |  |  |  |  |  |  |  |  |  |  |
| Upper secondary or vocational training | 1.480 | 6.39E-09 | 0.433 | 1.92E-34 | 0.458 | 1.10E-35 | 0.227 | 1.95E-10 | 0.179 | 1.86E-07 | 0.354 | 4.51E-15 | 0.377 | 1.81E-20 |
| Tertiary | 2.392 | 5.79E-07 | 0.843 | 8.10E-37 | 0.893 | 3.11E-38 | 0.361 | 7.24E-08 | 0.327 | 4.02E-07 | 0.787 | 2.12E-20 | 0.583 | 2.05E-14 |
| Cannot read or write | -3.383 | 1.52E-50 | -0.771 | 3.86E-127 | -0.660 | 3.78E-90 | -0.580 | 2.33E-73 | -0.732 | 1.04E-119 | -0.500 | 2.39E-36 | -0.563 | 4.27E-55 |
| Rural village | -0.330 | 0.112 | -0.104 | 2.45E-04 | -0.118 | 6.97E-05 | -0.071 | 0.015 | -0.031 | 0.263 | -0.106 | 0.004 | -0.052 | 0.114 |
| Scheduled caste | -0.937 | 0.003 | -0.092 | 3.32E-02 | -0.101 | 0.024 | -0.138 | 0.002 | -0.085 | 0.046 | -0.012 | 0.834 | -0.020 | 0.683 |
| Scheduled tribe | -1.715 | 0.001 | -0.152 | 3.27E-02 | -0.141 | 0.057 | -0.252 | 0.001 | -0.268 | 0.000 | -0.014 | 0.882 | 0.113 | 0.171 |
| Other backward class (obc) | -0.393 | 0.081 | -0.027 | 3.77E-01 | -0.024 | 0.457 | -0.060 | 0.057 | -0.035 | 0.248 | 0.007 | 0.850 | -0.016 | 0.662 |
| Per capita household consumption |  |  |  |  |  |  |  |  |  |  |  |  |  |  |
| Q2 | 0.353 | 0.179 | 0.054 | 1.31E-01 | 0.063 | 0.091 | 0.043 | 0.244 | 0.007 | 0.835 | 0.024 | 0.603 | 0.036 | 0.383 |
| Q3 | 0.727 | 0.007 | 0.086 | 1.97E-02 | 0.095 | 0.013 | 0.076 | 0.045 | -0.016 | 0.661 | 0.038 | 0.421 | 0.110 | 0.010 |
| Q4 | 0.862 | 0.002 | 0.129 | 6.44E-04 | 0.119 | 0.002 | 0.123 | 0.001 | 0.026 | 0.480 | 0.123 | 0.012 | 0.109 | 0.012 |
| Q5 | 0.544 | 0.057 | 0.106 | 6.58E-03 | 0.099 | 0.015 | 0.078 | 0.053 | 0.036 | 0.345 | 0.119 | 0.018 | 0.060 | 0.183 |
| **Total R^2^** | **0.421** | | **0.624** | | **0.582** | | **0.477** | | **0.525** | | **0.403** | | **0.379** | |

Abbreviations: APOE = apolipoprotein E; PC = principal component; HMSE = Hindi Mental State Examination.

## Supplementary Table 8. Two-way interaction between *APOE* ε4 carrier and educational level on cognitive measures.

|  | **Model 1 (n = 2,563)** | | **Model 2 (n = 2,548)** | |
| --- | --- | --- | --- | --- |
|  | **Beta** | **P value** | **Beta** | **P value** |
| **HMSE score** |  |  |  |  |
| *APOE* ε4 | **-0.872** | **4.25E-04** | **-0.770** | **0.001** |
| Upper secondary or vocational training | **3.498** | **7.97E-43** | **1.453** | **7.29E-08** |
| Tertiary | **4.481** | **8.71E-18** | **2.235** | **1.58E-05** |
| *APOE* ε4*Upper secondary or vocational training | 0.312 | 0.588 | 0.160 | 0.772 |
| *APOE* ε4*Tertiary | 0.878 | 0.474 | 0.931 | 0.424 |
| **General cognitive function** |  |  |  |  |
| *APOE* ε4 | **-0.100** | **0.006** | **-0.082** | **0.011** |
| Upper secondary or vocational training | **0.889** | **3.73E-115** | **0.432** | **6.40E-31** |
| Tertiary | **1.334** | **2.51E-64** | **0.827** | **1.01E-30** |
| *APOE* ε4*Upper secondary or vocational training | 0.024 | 0.775 | 0.001 | 0.986 |
| *APOE* ε4*Tertiary | 0.081 | 0.653 | 0.095 | 0.551 |
| **Executive function** |  |  |  |  |
| *APOE* ε4 | **-0.075** | **0.040** | -0.059 | 0.078 |
| Upper secondary or vocational training | **0.855** | **7.44E-107** | **0.460** | **2.46E-32** |
| Tertiary | **1.316** | **1.82E-62** | **0.873** | **1.11E-31** |
| *APOE* ε4*Upper secondary or vocational training | 0.010 | 0.907 | -0.013 | 0.867 |
| *APOE* ε4*Tertiary | 0.109 | 0.550 | 0.121 | 0.465 |
| **Orientation** |  |  |  |  |
| *APOE* ε4 | **-0.110** | **0.002** | **-0.091** | **0.006** |
| Upper secondary or vocational training | **0.585** | **1.09E-56** | **0.236** | **4.18E-10** |
| Tertiary | **0.724** | **5.06E-22** | **0.336** | **3.53E-06** |
| *APOE* ε4*Upper secondary or vocational training | -0.029 | 0.723 | -0.057 | 0.460 |
| *APOE* ε4*Tertiary | 0.141 | 0.423 | 0.150 | 0.359 |
| **Language/fluency** |  |  |  |  |
| *APOE* ε4 | -0.062 | 0.082 | -0.048 | 0.128 |
| Upper secondary or vocational training | **0.605** | **1.54E-60** | **0.187** | **2.76E-07** |
| Tertiary | **0.751** | **1.27E-23** | **0.302** | **1.44E-05** |
| *APOE* ε4*Upper secondary or vocational training | -0.030 | 0.720 | -0.050 | 0.498 |
| *APOE* ε4*Tertiary | 0.137 | 0.437 | 0.146 | 0.351 |
| **Memory** |  |  |  |  |
| *APOE* ε4 | **-0.092** | **0.033** | -0.081 | 0.051 |
| Upper secondary or vocational training | **0.670** | **3.51E-51** | **0.361** | **4.32E-14** |
| Tertiary | **1.132** | **3.79E-35** | **0.775** | **3.33E-17** |
| *APOE* ε4*Upper secondary or vocational training | -0.042 | 0.673 | -0.044 | 0.647 |
| *APOE* ε4*Tertiary | 0.063 | 0.770 | 0.073 | 0.722 |
| **Visuospatial function** |  |  |  |  |
| *APOE* ε4 | -0.056 | 0.156 | -0.048 | 0.195 |
| Upper secondary or vocational training | **0.675** | **3.77E-61** | **0.349** | **4.52E-16** |
| Tertiary | **0.942** | **1.42E-29** | **0.588** | **1.00E-12** |
| *APOE* ε4*Upper secondary or vocational training | **0.183** | **0.046*** | **0.175** | **0.045*** |
| *APOE* ε4*Tertiary | -0.049 | 0.803 | -0.030 | 0.872 |

Abbreviations: APOE = apolipoprotein E; LASI-DAD = Diagnostic Assessment of Dementia for the Longitudinal Aging Study of India; HMSE = Hindi Mental State Examination.

Model 1 adjusted for age, sex (male), state of residence, top 10 genetic PCs, education (upper secondary or vocational training and tertiary education), *APOE* ε4 × upper secondary or vocational training education, and *APOE* ε4 × upper secondary or tertiary education.

Model 2 adjusted for age, sex (male), state of residence, top 10 genetic PCs, education (upper secondary or vocational training and tertiary education), literacy, urban/rural residence, caste, quintiles of per capita household consumption, *APOE* ε4 × upper secondary or vocational training education, and *APOE* ε4 × upper secondary or tertiary education.

Beta coefficient and p-value in bold indicates statistically significant association at p<0.05.

Asterisks (*) indicates significant *APOE* ε4 by education interaction terms.

## Supplementary Table 9. Associations between *APOE* ε4 carrier and cognitive measures stratified by education level.

|  | Model 1 | | | | | | Model 2 | | | | | |
| --- | --- | --- | --- | --- | --- | --- | --- | --- | --- | --- | --- | --- |
|  | **Less than lower secondary (n=1,926)** | | **Upper secondary or vocational training (n=542)** | | **Tertiary**  **(n=95)** | | **Less than lower secondary (n=1,914)** | | **Upper secondary or vocational training (n=539)** | | **Tertiary**  **(n=95)** | |
| **Cognitive measure** | **Beta** | **P value** | **Beta** | **P value** | **Beta** | **P value** | **Beta** | **P value** | **Beta** | **P value** | **Beta** | **P value** |
| **HMSE score** | **-0.861** | **0.001** | -0.744 | 0.050 | -0.025 | 0.963 | **-0.758** | **0.003** | **-0.756** | **0.049** | 0.041 | 0.944 |
| **General cognitive function** | **-0.102** | **0.005** | -0.110 | 0.162 | 0.104 | 0.502 | **-0.082** | **0.008** | -0.110 | 0.156 | 0.120 | 0.471 |
| **Executive function** | **-0.076** | **0.037** | -0.096 | 0.231 | 0.186 | 0.309 | -0.059 | 0.071 | -0.093 | 0.240 | 0.190 | 0.335 |
| **Orientation** | **-0.112** | **0.003** | **-0.149** | **0.014** | 0.002 | 0.978 | **-0.093** | **0.007** | **-0.159** | **0.009** | -0.006 | 0.936 |
| **Language/fluency** | -0.061 | 0.096 | -0.091 | 0.187 | 0.034 | 0.819 | -0.045 | 0.161 | -0.093 | 0.161 | -0.006 | 0.972 |
| **Memory** | **-0.096** | **0.020** | -0.161 | 0.127 | 0.117 | 0.614 | **-0.081** | **0.038** | -0.155 | 0.142 | 0.096 | 0.704 |
| **Visuospatial function** | -0.055 | 0.150 | 0.089 | 0.334 | -0.056 | 0.811 | -0.047 | 0.189 | 0.087 | 0.346 | 0.047 | 0.852 |

Abbreviations: APOE = apolipoprotein E; LASI-DAD = Diagnostic Assessment of Dementia for the Longitudinal Aging Study of India; HMSE = Hindi Mental State Examination.

Model 1 adjusted for age, sex (male), state of residence, top 10 genetic PCs.

Model 2 adjusted for age, sex (male), state of residence, top 10 genetic PCs, literacy, urban/rural residence, caste, and quintiles of per capita household consumption.

Beta coefficient and p-value in bold indicates statistically significant association at p<0.05.

## Supplementary Table 10. Two-way interaction between *APOE* ε4 carrier and dichotomous education level on cognitive measures.

|  | **Model 1 (n = 2,563)** | | **Model 2 (n = 2,548)** | |
| --- | --- | --- | --- | --- |
|  | **Beta** | **P value** | **Beta** | **P value** |
| **HMSE score** |  |  |  |  |
| *APOE* ε4 | **-0.986** | **0.001** | **-0.880** | **0.002** |
| > 0 years of education | **4.396** | **4.79E-87** | **2.576** | **4.06E-22** |
| *APOE* ε4 × > 0 years of education | 0.410 | 0.330 | 0.346 | 0.401 |
| **General cognitive function** |  |  |  |  |
| *APOE* ε4 | **-0.121** | **0.008** | **-0.102** | **0.013** |
| > 0 years of education | **0.899** | **2.33E-144** | **0.360** | **2.62E-21** |
| *APOE* ε4 × > 0 years of education | 0.035 | 0.588 | 0.034 | 0.568 |
| **Executive function** |  |  |  |  |
| *APOE* ε4 | -0.083 | 0.070 | -0.066 | 0.122 |
| > 0 years of education | **0.844** | **4.51E-126** | **0.369** | **8.96E-21** |
| *APOE* ε4 × > 0 years of education | 0.004 | 0.956 | 0.003 | 0.965 |
| **Orientation** |  |  |  |  |
| *APOE* ε4 | **-0.114** | **0.007** | **-0.094** | **0.020** |
| > 0 years of education | **0.701** | **2.50E-105** | **0.362** | **3.60E-22** |
| *APOE* ε4 × > 0 years of education | 0.005 | 0.928 | -0.006 | 0.913 |
| **Language/fluency** |  |  |  |  |
| *APOE* ε4 | **-0.091** | **0.033** | -0.076 | 0.052 |
| > 0 years of education | **0.650** | **2.44E-89** | **0.163** | **6.69E-06** |
| *APOE* ε4 × > 0 years of education | 0.051 | 0.403 | 0.045 | 0.429 |
| **Memory** |  |  |  |  |
| *APOE* ε4 | **-0.135** | **0.013** | **-0.126** | **0.016** |
| > 0 years of education | **0.602** | **1.52E-50** | **0.205** | **2.00E-05** |
| *APOE* ε4 × > 0 years of education | 0.056 | 0.475 | 0.067 | 0.373 |
| **Visuospatial function** |  |  |  |  |
| *APOE* ε4 | -0.056 | 0.247 | -0.050 | 0.286 |
| > 0 years of education | **0.692** | **8.06E-80** | **0.319** | **1.36E-13** |
| *APOE* ε4 × > 0 years of education | 0.047 | 0.495 | 0.053 | 0.433 |

Abbreviations: APOE = apolipoprotein E; LASI-DAD = Diagnostic Assessment of Dementia for the Longitudinal Aging Study of India; HMSE = Hindi Mental State Examination.

Model 1 adjusted for age, sex (male), state of residence, top 10 genetic PCs, education (> 0 years of education), and *APOE* ε4 × > 0 years of education.

Model 2 adjusted for age, sex (male), state of residence, top 10 genetic PCs, education (> 0 years of education), literacy, urban/rural residence, caste, quintiles of per capita household consumption, and *APOE* ε4 × > 0 years of education.

Beta coefficient and p-value in bold indicates statistically significant association at p<0.05.

## Supplementary Table 11. Associations between *APOE* ε4 carrier and cognitive measures stratified by education (dichotomized at having received some formal schooling).

|  | Model 1 | | | | Model 2 | | | |
| --- | --- | --- | --- | --- | --- | --- | --- | --- |
|  | **0 years (n = 1,249)** | | **>0 years  (n = 1,314)** | | **0 years  (n = 1,239)** | | **>0 years  (n = 1,348)** | |
| **Cognitive measure** | **Beta** | **P value** | **Beta** | **P value** | **Beta** | **P value** | **Beta** | **P value** |
| **HMSE score** | **-0.902** | **0.005** | **-0.695** | **0.011** | **-0.779** | **0.016** | **-0.630** | **0.016** |
| **General cognitive function** | **-0.122** | **0.002** | -0.100 | 0.056 | **-0.101** | **0.006** | -0.076 | 0.099 |
| **Executive function** | **-0.084** | **0.031** | -0.089 | 0.094 | -0.065 | 0.084 | -0.067 | 0.170 |
| **Orientation** | **-0.114** | **0.008** | **-0.116** | **0.007** | **-0.089** | **0.034** | **-0.105** | **0.009** |
| **Language/fluency** | **-0.088** | **0.035** | -0.048 | 0.298 | -0.070 | 0.085 | -0.036 | 0.368 |
| **Memory** | **-0.141** | **0.003** | -0.096 | 0.120 | **-0.129** | **0.006** | -0.073 | 0.216 |
| **Visuospatial function** | -0.057 | 0.167 | -0.025 | 0.663 | -0.052 | 0.203 | -0.005 | 0.922 |

Abbreviations: APOE = apolipoprotein E; LASI-DAD = Diagnostic Assessment of Dementia for the Longitudinal Aging Study of India; HMSE = Hindi Mental State Examination.

Model 1 adjusted for age, sex (male), state of residence, top 10 genetic PCs.

Model 2 adjusted for age, sex (male), state of residence, top 10 genetic PCs, literacy, urban/rural residence, caste, and quintiles of per capita household consumption.

Beta coefficient and p-value in bold indicates statistically significant association at p<0.05.

## Supplementary Table 12. Main effects of *APOE* genotypes on cognitive measures in LASI-DAD.

|  | **Model 1 (n = 2,558)** | | **Model 2 (n = 2,543)** | |
| --- | --- | --- | --- | --- |
|  | **Beta** | **P value** | **Beta** | **P value** |
| **HMSE score** |  |  |  |  |
| ε3/ε3 | ref | ref | ref | ref |
| ε2/ε3 | 0.181 | 0.589 | 0.009 | 0.977 |
| ε3/ε4 | **-0.969** | **5.99E-05** | **-0.727** | **0.001** |
| ε4/ε4 | -0.818 | 0.293 | -0.495 | 0.481 |
| **General cognitive function** |  |  |  |  |
| ε3/ε3 | ref | ref | ref | ref |
| ε2/ε3 | 0.006 | 0.916 | -0.039 | 0.349 |
| ε3/ε4 | **-0.140** | **3.61E-04** | **-0.083** | **0.005** |
| ε4/ε4 | -0.150 | 0.237 | -0.079 | 0.414 |
| **Executive function** |  |  |  |  |
| ε3/ε3 | ref | ref | ref | ref |
| ε2/ε3 | 0.001 | 0.989 | -0.038 | 0.381 |
| ε3/ε4 | **-0.113** | **0.004** | -0.060 | 0.055 |
| ε4/ε4 | -0.162 | 0.200 | -0.083 | 0.406 |
| **Orientation** |  |  |  |  |
| ε3/ε3 | ref | ref | ref | ref |
| ε2/ε3 | -0.015 | 0.756 | -0.053 | 0.214 |
| ε3/ε4 | **-0.143** | **4.90E-05** | **-0.102** | **0.001** |
| ε4/ε4 | -0.139 | 0.220 | -0.094 | 0.339 |
| **Language/fluency** |  |  |  |  |
| ε3/ε3 | ref | ref | ref | ref |
| ε2/ε3 | 0.092 | 0.059 | 0.060 | 0.142 |
| ε3/ε4 | **-0.090** | **0.011** | -0.050 | 0.092 |
| ε4/ε4 | -0.018 | 0.871 | -0.001 | 0.992 |
| **Memory** |  |  |  |  |
| ε3/ε3 | ref | ref | ref | ref |
| ε2/ε3 | 0.015 | 0.799 | -0.015 | 0.783 |
| ε3/ε4 | **-0.130** | **0.002** | **-0.088** | **0.023** |
| ε4/ε4 | -0.170 | 0.216 | -0.102 | 0.410 |
| **Visuospatial function** |  |  |  |  |
| ε3/ε3 | ref | ref | ref | ref |
| ε2/ε3 | -0.080 | 0.146 | **-0.119** | **0.014** |
| ε3/ε4 | -0.075 | 0.057 | -0.035 | 0.307 |
| ε4/ε4 | -0.022 | 0.863 | 0.020 | 0.861 |

Abbreviations: APOE = apolipoprotein E; LASI-DAD = Diagnostic Assessment of Dementia for the Longitudinal Aging Study of India; HMSE = Hindi Mental State Examination; ref = referent.

Model 1 adjusted for age, sex (male), state of residence, and top 10 genetic PCs.

Model 2 adjusted for age, sex (male), state of residence, top 10 genetic PCs, education (less than lower secondary, upper secondary or vocational training and tertiary education), literacy, urban/rural residence, caste, and quintiles of per capita household consumption.

## Supplementary Table 13. Two-way interaction between *APOE* genotypes and continuous age on cognitive measures.

|  | **Model 1 (n = 2,558)** | | **Model 2 (n = 2,543)** | |
| --- | --- | --- | --- | --- |
|  | **Beta** | **P value** | **Beta** | **P value** |
| **HMSE score** |  |  |  |  |
| ε3/ε3*Age | ref | ref | ref | ref |
| ε2/ε3*Age | -0.009 | 0.848 | -0.004 | 0.926 |
| ε3/ε4*Age | -0.057 | 0.080 | **-0.071** | **0.016** |
| ε4/ε4*Age | 0.064 | 0.538 | 0.064 | 0.493 |
| **General cognitive function** |  |  |  |  |
| ε3/ε3*Age | ref | ref | ref | ref |
| ε2/ε3*Age | -0.004 | 0.595 | -0.004 | 0.457 |
| ε3/ε4*Age | -0.004 | 0.451 | **-0.008** | **0.042** |
| ε4/ε4*Age | 0.002 | 0.903 | 0.002 | 0.899 |
| **Executive function** |  |  |  |  |
| ε3/ε3*Age | ref | ref | ref | ref |
| ε2/ε3*Age | -1.78E-04 | 0.981 | -1.12E-04 | 0.985 |
| ε3/ε4*Age | -0.002 | 0.695 | -0.007 | 0.118 |
| ε4/ε4*Age | 0.006 | 0.742 | 0.004 | 0.752 |
| **Orientation** |  |  |  |  |
| ε3/ε3*Age | ref | ref | ref | ref |
| ε2/ε3*Age | -0.006 | 0.328 | -0.007 | 0.210 |
| ε3/ε4*Age | **-0.010** | **0.044** | **-0.012** | **0.004** |
| ε4/ε4*Age | 0.004 | 0.802 | 0.004 | 0.744 |
| **Language/fluency** |  |  |  |  |
| ε3/ε3*Age | ref | ref | ref | ref |
| ε2/ε3*Age | -0.001 | 0.918 | -4.57E-04 | 0.935 |
| ε3/ε4*Age | -0.008 | 0.078 | **-0.010** | **0.009** |
| ε4/ε4*Age | -4.14E-04 | 0.978 | 2.46E-04 | 0.984 |
| **Memory** |  |  |  |  |
| ε3/ε3*Age | ref | ref | ref | ref |
| ε2/ε3*Age | -0.010 | 0.209 | -0.011 | 0.137 |
| ε3/ε4*Age | -0.001 | 0.895 | -0.004 | 0.433 |
| ε4/ε4*Age | 0.004 | 0.833 | 0.003 | 0.846 |
| **Visuospatial function** |  |  |  |  |
| ε3/ε3*Age | ref | ref | ref | ref |
| ε2/ε3*Age | -0.001 | 0.873 | -0.002 | 0.807 |
| ε3/ε4*Age | 0.004 | 0.494 | 7.68E-05 | 0.987 |
| ε4/ε4*Age | -0.014 | 0.421 | -0.013 | 0.368 |

Abbreviations: APOE = apolipoprotein E; LASI-DAD = Diagnostic Assessment of Dementia for the Longitudinal Aging Study of India; HMSE = Hindi Mental State Examination; ref = referent.

Model 1 adjusted for age (age > 68), sex (male), state of residence, top 10 genetic PCs, and *APOE* × Age > 68.

Model 2 adjusted for age (age > 68), sex (male), state of residence, top 10 genetic PCs, education (less than lower secondary, upper secondary or vocational training and tertiary education), literacy, urban/rural residence, caste, quintiles of per capita household consumption, and *APOE* × Age > 68.

Beta coefficient and p-value in bold indicates statistically significant association at p<0.05.

Asterisk (*) denotes cognitive measures for which a statistically significant interaction term was observed.

## Supplementary Table 14. Two-way interaction between *APOE* genotypes and sex on cognitive measures.

|  | **Model 1 (n = 2,558)** | | **Model 2 (n = 2,543)** | |
| --- | --- | --- | --- | --- |
|  | **Beta** | **P value** | **Beta** | **P value** |
| **HMSE score** |  |  |  |  |
| ε3/ε3*Male | ref | ref | ref | ref |
| ε2/ε3*Male | **1.446** | **0.031** | **1.420** | **0.020** |
| ε3/ε4*Male | 0.785 | 0.103 | 0.681 | 0.119 |
| ε4/ε4*Male | 2.334 | 0.133 | 1.552 | 0.269 |
| **General cognitive function** |  |  |  |  |
| ε3/ε3*Male | ref | ref | ref | ref |
| ε2/ε3*Male | 0.071 | 0.513 | 0.068 | 0.417 |
| ε3/ε4*Male | 0.106 | 0.179 | 0.082 | 0.171 |
| ε4/ε4*Male | 0.420 | 0.098 | 0.273 | 0.156 |
| **Executive function** |  |  |  |  |
| ε3/ε3*Male | ref | ref | ref | ref |
| ε2/ε3*Male | 0.065 | 0.546 | 0.074 | 0.393 |
| ε3/ε4*Male | 0.009 | 0.907 | -0.010 | 0.872 |
| ε4/ε4*Male | 0.308 | 0.222 | 0.182 | 0.362 |
| **Orientation** |  |  |  |  |
| ε3/ε3*Male | ref | ref | ref | ref |
| ε2/ε3*Male | 0.088 | 0.367 | 0.085 | 0.321 |
| ε3/ε4*Male | 0.130 | 0.064 | 0.109 | 0.075 |
| ε4/ε4*Male | 0.271 | 0.232 | 0.143 | 0.467 |
| **Language/fluency*** |  |  |  |  |
| ε3/ε3*Male | ref | ref | ref | ref |
| ε2/ε3*Male | 0.086 | 0.380 | 0.068 | 0.407 |
| ε3/ε4*Male | **0.139** | **0.048** | **0.119** | **0.043** |
| ε4/ε4*Male | 0.404 | 0.075 | 0.247 | 0.191 |
| **Memory*** |  |  |  |  |
| ε3/ε3*Male | ref | ref | ref | ref |
| ε2/ε3*Male | -0.093 | 0.432 | -0.093 | 0.390 |
| ε3/ε4*Male | 0.161 | 0.059 | **0.152** | **0.049** |
| ε4/ε4*Male | 0.371 | 0.178 | 0.286 | 0.248 |
| **Visuospatial function** |  |  |  |  |
| ε3/ε3*Male | ref | ref | ref | ref |
| ε2/ε3*Male | 0.186 | 0.089 | 0.162 | 0.093 |
| ε3/ε4*Male | 0.056 | 0.481 | 0.032 | 0.645 |
| ε4/ε4*Male | 0.322 | 0.206 | 0.248 | 0.265 |

Abbreviations: APOE = apolipoprotein E; LASI-DAD = Diagnostic Assessment of Dementia for the Longitudinal Aging Study of India; HMSE = Hindi Mental State Examination; ref = referent.

Model 1 adjusted for age, sex (male), state of residence, top 10 genetic PCs, and *APOE* × Male.

Model 2 adjusted for age, sex (male), state of residence, top 10 genetic PCs, education (less than lower secondary, upper secondary or vocational training and tertiary education), literacy, urban/rural residence, caste, quintiles of per capita household consumption, and *APOE* × Male.

Beta coefficient and p-value in bold indicates statistically significant association at p<0.05.

Asterisk (*) denotes cognitive measures for which a statistically significant interaction term was observed.

## Supplementary Table 15. Associations between *APOE* genotypes and cognitive measures stratified by sex.

|  | Model 1 | | | | Model 2 | | | |
| --- | --- | --- | --- | --- | --- | --- | --- | --- |
|  | **Male (n = 1,203)** | | **Female (n = 1,355)** | | **Male (n = 1,198)** | | **Female (n = 1,345)** | |
| **Cognitive Measure** | **Beta** | **P value** | **Beta** | **P value** | **Beta** | **P value** | **Beta** | **P value** |
| **HMSE score** |  |  |  |  |  |  |  |  |
| ε3/ε3 | ref | ref | ref | ref | ref | ref | ref | ref |
| ε2/ε3 | **0.904** | **0.049** | -0.468 | 0.335 | 0.704 | 0.089 | -0.668 | 0.138 |
| ε3/ε4 | -0.498 | 0.138 | **-1.236** | **3.48E-04** | -0.370 | 0.218 | **-0.941** | **0.003** |
| ε4/ε4 | 0.470 | 0.656 | -2.093 | 0.067 | 0.324 | 0.731 | -1.317 | 0.208 |
| **General cognitive function** |  |  |  |  |  |  |  |  |
| ε3/ε3 | ref | ref | ref | ref | ref | ref | ref | ref |
| ε2/ε3 | 0.040 | 0.626 | -0.017 | 0.821 | -0.010 | 0.868 | -1.317 | 0.208 |
| ε3/ε4 | -0.079 | 0.181 | **-0.176** | **0.001** | -0.045 | 0.309 | -0.064 | 0.269 |
| ε4/ε4 | 0.075 | 0.688 | **-0.377** | **0.030** | 0.049 | 0.725 | **-0.112** | **0.006** |
| **Executive function** |  |  |  |  |  |  |  |  |
| ε3/ε3 | ref | ref | ref | ref | ref | ref | ref | ref |
| ε2/ε3 | 0.027 | 0.743 | -0.017 | 0.809 | -0.008 | 0.898 | -0.059 | 0.318 |
| ε3/ε4 | -0.103 | 0.090 | **-0.106** | **0.038** | -0.066 | 0.167 | -0.049 | 0.237 |
| ε4/ε4 | 0.006 | 0.976 | -0.310 | 0.067 | -0.005 | 0.974 | -0.181 | 0.185 |
| **Orientation** |  |  |  |  |  |  |  |  |
| ε3/ε3 | ref | ref | ref | ref | ref | ref | ref | ref |
| ε2/ε3 | 0.032 | 0.634 | -0.048 | 0.492 | -0.010 | 0.863 | -0.093 | 0.133 |
| ε3/ε4 | -0.059 | 0.231 | **-0.201** | **5.06E-05** | -0.043 | 0.311 | **-0.152** | **4.75E-04** |
| ε4/ε4 | 0.030 | 0.848 | -0.302 | 0.066 | -0.003 | 0.983 | -0.176 | 0.220 |
| **Language/fluency*** |  |  |  |  |  |  |  |  |
| ε3/ε3 | ref | ref | ref | ref | ref | ref | ref | ref |
| ε2/ε3 | 0.126 | 0.081 | 0.048 | 0.469 | 0.081 | 0.176 | 0.021 | 0.714 |
| ε3/ε4 | -0.014 | 0.796 | **-0.136** | **0.004** | 0.008 | 0.861 | **-0.088** | **0.029** |
| ε4/ε4 | 0.174 | 0.295 | -0.228 | 0.146 | 0.106 | 0.437 | -0.120 | 0.368 |
| **Memory*** |  |  |  |  |  |  |  |  |
| ε3/ε3 | ref | ref | ref | ref | ref | ref | ref | ref |
| ε2/ε3 | -0.031 | 0.719 | 0.067 | 0.417 | -0.061 | 0.430 | 0.025 | 0.736 |
| ε3/ε4 | -0.047 | 0.452 | **-0.187** | **0.001** | -0.023 | 0.681 | **-0.142** | **0.007** |
| ε4/ε4 | 0.031 | 0.874 | -0.373 | 0.054 | 0.021 | 0.905 | -0.242 | 0.166 |
| **Visuospatial function** |  |  |  |  |  |  |  |  |
| ε3/ε3 | ref | ref | ref | ref | ref | ref | ref | ref |
| ε2/ε3 | 0.023 | 0.783 | **-0.150** | **0.041** | -0.030 | 0.676 | **-0.176** | **0.007** |
| ε3/ε4 | -0.053 | 0.378 | -0.092 | 0.079 | -0.026 | 0.623 | -0.042 | 0.362 |
| ε4/ε4 | 0.139 | 0.460 | -0.215 | 0.213 | 0.150 | 0.359 | -0.143 | 0.349 |

Abbreviations: APOE = apolipoprotein E; HMSE = Hindi Mental State Examination; ref = referent.

Model 1 adjusted for age, state of residence, and top 10 genetic PCs.

Model 2 adjusted for age, state of residence, top 10 genetic PCs, education (less than lower secondary, upper secondary or vocational training and tertiary education), literacy, urban/rural residence, caste, and quintiles of per capita household consumption.

Beta coefficient and p-value in bold indicates statistically significant association at p<0.05.

Asterisk (*) denotes cognitive measures for which a statistically significant interaction term was observed.

## Supplementary Table 16. Two-way interaction between *APOE* genotypes and educational level (dichotomized at having received some formal schooling) on cognitive measures.

|  | **Model 1 (n = 2,558)** | | **Model 2 (n = 2,543)** | |
| --- | --- | --- | --- | --- |
|  | **Beta** | **P value** | **Beta** | **P value** |
| **HMSE score** |  |  |  |  |
| ε3/ε3 × > 0 years of education | ref | ref | ref | ref |
| ε2/ε3 × > 0 years of education | 0.067 | 0.913 | 0.137 | 0.821 |
| ε3/ε4 × > 0 years of education | 0.455 | 0.301 | 0.419 | 0.330 |
| ε4/ε4 × > 0 years of education | -0.070 | 0.961 | -0.467 | 0.740 |
| **General cognitive function** |  |  |  |  |
| ε3/ε3 × > 0 years of education | ref | ref | ref | ref |
| ε2/ε3 × > 0 years of education | 0.014 | 0.880 | 0.022 | 0.800 |
| ε3/ε4 × > 0 years of education | 0.052 | 0.438 | 0.055 | 0.370 |
| ε4/ε4 × > 0 years of education | -0.177 | 0.425 | -0.245 | 0.221 |
| **Executive function** |  |  |  |  |
| ε3/ε3 × > 0 years of education | ref | ref | ref | ref |
| ε2/ε3 × > 0 years of education | 0.038 | 0.692 | 0.053 | 0.554 |
| ε3/ε4 × > 0 years of education | 0.017 | 0.803 | 0.021 | 0.741 |
| ε4/ε4 × > 0 years of education | -0.123 | 0.584 | -0.189 | 0.366 |
| **Orientation** |  |  |  |  |
| ε3/ε3 × > 0 years of education | ref | ref | ref | ref |
| ε2/ε3 × > 0 years of education | -0.070 | 0.423 | -0.057 | 0.502 |
| ε3/ε4 × > 0 years of education | 3.18E-04 | 0.996 | -0.008 | 0.897 |
| ε4/ε4 × > 0 years of education | -0.035 | 0.866 | -0.086 | 0.662 |
| **Language/fluency** |  |  |  |  |
| ε3/ε3 × > 0 years of education | ref | ref | ref | ref |
| ε2/ε3 × > 0 years of education | 0.018 | 0.843 | 0.005 | 0.953 |
| ε3/ε4 × > 0 years of education | 0.059 | 0.356 | 0.052 | 0.381 |
| ε4/ε4 × > 0 years of education | -0.012 | 0.955 | -0.053 | 0.782 |
| **Memory** |  |  |  |  |
| ε3/ε3 × > 0 years of education | ref | ref | ref | ref |
| ε2/ε3 × > 0 years of education | 0.036 | 0.750 | 0.049 | 0.658 |
| ε3/ε4 × > 0 years of education | 0.087 | 0.282 | 0.103 | 0.189 |
| ε4/ε4 × > 0 years of education | -0.340 | 0.200 | -0.387 | 0.130 |
| **Visuospatial function** |  |  |  |  |
| ε3/ε3 × > 0 years of education | ref | ref | ref | ref |
| ε2/ε3 × > 0 years of education | -0.036 | 0.722 | -0.047 | 0.633 |
| ε3/ε4 × > 0 years of education | 0.071 | 0.330 | 0.077 | 0.269 |
| ε4/ε4 × > 0 years of education | -0.294 | 0.215 | -0.336 | 0.141 |

Abbreviations: APOE = apolipoprotein E; LASI-DAD = Diagnostic Assessment of Dementia for the Longitudinal Aging Study of India; HMSE = Hindi Mental State Examination; ref = referent.

Model 1 adjusted for age, sex (male), state of residence, top 10 genetic PCs, education (> 0 years of education), and *APOE* × > 0 years of education.

Model 2 adjusted for age, sex (male), state of residence, top 10 genetic PCs, education (> 0 years of education), literacy, urban/rural residence, caste, quintiles of per capita household consumption, and *APOE* × > 0 years of education.

Beta coefficient and p-value in bold indicates statistically significant association at p<0.05.

## Supplementary Table 17. Associations between *APOE* genotype and cognitive measures stratified by education level (dichotomized at having received some formal schooling).

|  | Model 1 | | | | Model 2 | | | |
| --- | --- | --- | --- | --- | --- | --- | --- | --- |
|  | **0 years (n = 1,247)** | | **>0 years  (n = 1,311)** | | **0 years (n = 1,239)** | | **>0 years  (n = 1,306)** | |
| **Cognitive Measure** | **Beta** | **P value** | **Beta** | **P value** | **Beta** | **P value** | **Beta** | **P value** |
| **HMSE score** |  |  |  |  |  |  |  |  |
| ε3/ε3 | ref | ref | ref | ref | ref | ref | ref | ref |
| ε2/ε3 | 0.104 | 0.836 | 0.145 | 0.696 | -0.072 | 0.886 | 0.117 | 0.746 |
| ε3/ε4 | **-0.934** | **0.006** | **-0.685** | **0.015** | **-0.819** | **0.015** | **-0.605** | **0.026** |
| ε4/ε4 | -0.350 | 0.734 | -0.628 | 0.524 | -0.339 | 0.740 | -0.858 | 0.365 |
| **General cognitive function** |  |  |  |  |  |  |  |  |
| ε3/ε3 | ref | ref | ref | ref | ref | ref | ref | ref |
| ε2/ε3 | -0.019 | 0.746 | 0.008 | 0.915 | -0.054 | 0.355 | -0.020 | 0.747 |
| ε3/ε4 | **-0.136** | **0.001** | -0.095 | 0.081 | **-0.113** | **0.004** | -0.067 | 0.164 |
| ε4/ε4 | 0.021 | 0.865 | -0.172 | 0.362 | -0.020 | 0.867 | -0.267 | 0.108 |
| **Executive function** |  |  |  |  |  |  |  |  |
| ε3/ε3 | ref | ref | ref | ref | ref | ref | ref | ref |
| ε2/ε3 | -0.033 | 0.579 | 0.010 | 0.890 | -0.064 | 0.282 | -0.012 | 0.862 |
| ε3/ε4 | -0.094 | 0.021 | -0.083 | 0.131 | -0.074 | 0.063 | -0.056 | 0.264 |
| ε4/ε4 | -0.003 | 0.981 | -0.161 | 0.404 | -0.036 | 0.765 | -0.246 | 0.161 |
| **Orientation** |  |  |  |  |  |  |  |  |
| ε3/ε3 | ref | ref | ref | ref | ref | ref | ref | ref |
| ε2/ε3 | 0.012 | 0.857 | -0.046 | 0.425 | -0.028 | 0.672 | -0.062 | 0.265 |
| ε3/ε4 | **-0.117** | **0.010** | **-0.124** | **0.005** | **-0.092** | **0.037** | **-0.112** | **0.008** |
| ε4/ε4 | -0.052 | 0.702 | -0.079 | 0.610 | -0.079 | 0.554 | -0.125 | 0.390 |
| **Language/fluency** |  |  |  |  |  |  |  |  |
| ε3/ε3 | ref | ref | ref | ref | ref | ref | ref | ref |
| ε2/ε3 | 0.065 | 0.311 | 0.092 | 0.142 | 0.052 | 0.413 | 0.060 | 0.272 |
| ε3/ε4 | **-0.092** | **0.035** | -0.044 | 0.356 | -0.071 | 0.094 | -0.029 | 0.483 |
| ε4/ε4 | 0.047 | 0.722 | 0.039 | 0.816 | 0.016 | 0.903 | -0.050 | 0.729 |
| **Memory** |  |  |  |  |  |  |  |  |
| ε3/ε3 | ref | ref | ref | ref | ref | ref | ref | ref |
| ε2/ε3 | -0.023 | 0.752 | 0.040 | 0.637 | -0.062 | 0.401 | 0.026 | 0.750 |
| ε3/ε4 | **-0.157** | **0.002** | -0.079 | 0.218 | **-0.145** | **0.003** | -0.052 | 0.395 |
| ε4/ε4 | 0.018 | 0.904 | -0.311 | 0.164 | -0.010 | 0.949 | -0.375 | 0.080 |
| **Visuospatial function** |  |  |  |  |  |  |  |  |
| ε3/ε3 | ref | ref | ref | ref | ref | ref | ref | ref |
| ε2/ε3 | -0.072 | 0.257 | -0.105 | 0.169 | -0.083 | 0.190 | -0.136 | 0.066 |
| ε3/ε4 | -0.085 | 0.052 | -0.029 | 0.626 | -0.076 | 0.076 | -0.007 | 0.904 |
| ε4/ε4 | 0.164 | 0.213 | -0.157 | 0.443 | 0.122 | 0.347 | -0.230 | 0.236 |

Abbreviations: APOE = apolipoprotein E; LASI-DAD = Diagnostic Assessment of Dementia for the Longitudinal Aging Study of India; HMSE = Hindi Mental State Examination; ref = referent.

Model 1 adjusted for age, sex (male), state of residence, top 10 genetic PCs.

Model 2 adjusted for age, sex (male), state of residence, top 10 genetic PCs, literacy, urban/rural residence, caste, and quintiles of per capita household consumption.

Beta coefficient and p-value in bold indicates statistically significant association at p<0.05.

## Supplementary Table 18. Significant associations between *APOE* ε4 carrier status and cognitive measures additionally adjusted for vascular risk factors in LASI-DAD.

|  | Model 2 + vascular risk factors (n = 2,355) | | |
| --- | --- | --- | --- |
| **Cognitive measure** | **Beta** | **P value** | **ΔR^2^** |
| **HMSE score** | **-0.413** | **0.027** | 0.12% |
| **General cognitive function** | **-0.050** | **0.049** | 0.06% |
| **Orientation** | **-0.063** | **0.017** | 0.12% |
| **Memory** | **-0.071** | **0.036** | 0.11% |
| Abbreviations: APOE = apolipoprotein E; LASI-DAD = Diagnostic Assessment of Dementia for the Longitudinal Aging Study of India; HMSE = Hindi Mental State Examination.  Model adjusted for age, sex, state of residence, top 10 genetic PCs, education (less than lower secondary, upper secondary or vocational training and tertiary education), literacy, urban/rural residence, caste, quintiles of per capita household consumption, high blood pressure, smoking (ever smoked), body mass index (normal, underweight, overweight/obese), diabetes, and high cholesterol.  ΔR^2^ represents the change in R^2^ when *APOE* ε4 carrier status was added to the corresponding regression models.  Beta coefficient and p-value in bold indicates statistically significant association at p<0.05. | | | |

## Supplementary Table 19. Significant *APOE* ε4 interactions after additionally adjusted for vascular risk factors in LASI-DAD.

|  |  | ***APOE* ε4 main effect** | | **Effect modifier main effect** | | **Interaction effect** | |
| --- | --- | --- | --- | --- | --- | --- | --- |
| **Cognitive measure** | **Interaction** | **Beta** | **P value** | **Beta** | **P value** | **Beta** | **P value** |
| HMSE score | *APOE* ε4 × Age | **-0.481** | **0.021** | **-0.125** | **2.01E-21** | **-0.061** | **0.034** |
| Orientation | *APOE* ε4 × Age | **-0.073** | **0.013** | **-0.014** | **5.06E-14** | **-0.009** | **0.021** |
| Language/fluency | *APOE* ε4 × Age | -0.029 | 0.309 | **-0.012** | **1.50E-11** | **-0.010** | **0.013** |
| Language/fluency | *APOE* ε4 × Male | -0.076 | 0.051 | **0.065** | **0.026** | 0.105 | 0.062 |
| Memory | *APOE* ε4 × Male | **-0.147** | **0.005** | **-0.080** | **0.042** | 0.147 | 0.052 |
| Visuospatial function | *APOE* ε4 × Upper secondary or vocational training | -0.039 | 0.308 | **0.389** | **2.77E-18** | **0.181** | **0.046** |
| Abbreviations: APOE = apolipoprotein E; LASI-DAD = Diagnostic Assessment of Dementia for the Longitudinal Aging Study of India; HMSE = Hindi Mental State Examination.  Model for testing *APOE* ε4 by age interaction adjusted for age (continuous, centered at the sample mean), sex, state of residence, top 10 genetic PCs, education (less than lower secondary, upper secondary or vocational training and tertiary education), literacy, urban/rural residence, caste, quintiles of per capita household consumption, high blood pressure, smoking (ever smoked), body mass index (normal, underweight, overweight/obese), diabetes, high cholesterol, and *APOE* ε4 × Age.  Model for testing *APOE* ε4 by sex interaction adjusted for age, sex (male), state of residence, top 10 genetic PCs, education (less than lower secondary, upper secondary or vocational training and tertiary education), literacy, urban/rural residence, caste, quintiles of per capita household consumption, high blood pressure, smoking (ever smoked), body mass index (normal, underweight, overweight/obese), diabetes, high cholesterol, and *APOE* ε4 × male.  Model for testing *APOE* ε4 by education interaction adjusted for age, sex (male), state of residence, top 10 genetic PCs, education (less than lower secondary, upper secondary or vocational training and tertiary education), literacy, urban/rural residence, caste, quintiles of per capita household consumption, *APOE* ε4 × upper secondary or vocational training education, *APOE* ε4 × upper secondary or tertiary education, high blood pressure, smoking (ever smoked), body mass index (normal, underweight, overweight/obese), diabetes, and high cholesterol.  Beta coefficient and p-value in bold indicates statistically significant association at p<0.05. | | | | | | | |
